# Au(I)-based compounds inhibit nsp14/nsp10 and nsp13 (helicase) to exert anti-SARS-CoV-2 properties

**DOI:** 10.1007/s00775-025-02118-9

**Published:** 2025-06-18

**Authors:** Jingxin Chen, Xueying Wei, Chun-Lung Chan, Kaiming Tang, Shuofeng Yuan, Hongyan Li, Hongzhe Sun

**Affiliations:** 1 Department of Chemistry and HKU-CAS Joint Laboratory of Metallomics on Health and Environment, The University of Hong Kong, Pokfulam Road, Hong Kong SAR, China; 2 Department of Microbiology and State Key Laboratory of Emerging Infectious Diseases, The University of Hong Kong, Pokfulam Road, Hong Kong SAR, China

**Keywords:** SARS-CoV-2, Antiviral, Metallodrug, nsp14, Helicase, Gold

## Abstract

Au(I) compounds have long been associated with medicine for the treatment of various diseases, especially auranofin has been used for the treatment of rheumatoid arthritis. In addition, Au(I) based compounds also exhibit anti-cancer, anti-bacteria properties. The recent prevalence of the COVID-19 pandemic, caused by severe acute respiratory syndrome coronavirus 2 (SARS-CoV-2), has aroused attention to investigate the antiviral potential of Au(I) compounds. Herein we demonstrate the pan-anti-SARS-CoV-2 activity of Au(I) metallodrugs in mammalian cells. We synthesized a panel of Au(I)-based compounds and found that these compounds could effectively inhibit the exoribonuclease and methyltransferase activities of SARS-CoV-2 nsp14/nsp10 complex, and the ATPase and DNA unwinding activities of SARS-CoV-2 nsp13 (helicase). Mechanistic studies reveal that Au(I) can not only displace the critical Zn(II) ions from nsp14/nsp10 complex and nsp13 but also changes the secondary and quaternary structure of nsp14 and perturbate the DNA unwinding of nsp13 by disrupting the ATP binding. This study illustrates a multi-target feature Au(I) compounds/drug agents for the viruses, highlighting their potential as pan-anti-SARS-CoV-2 (or relevant viruses) agents.

**Graphical Abstract:**

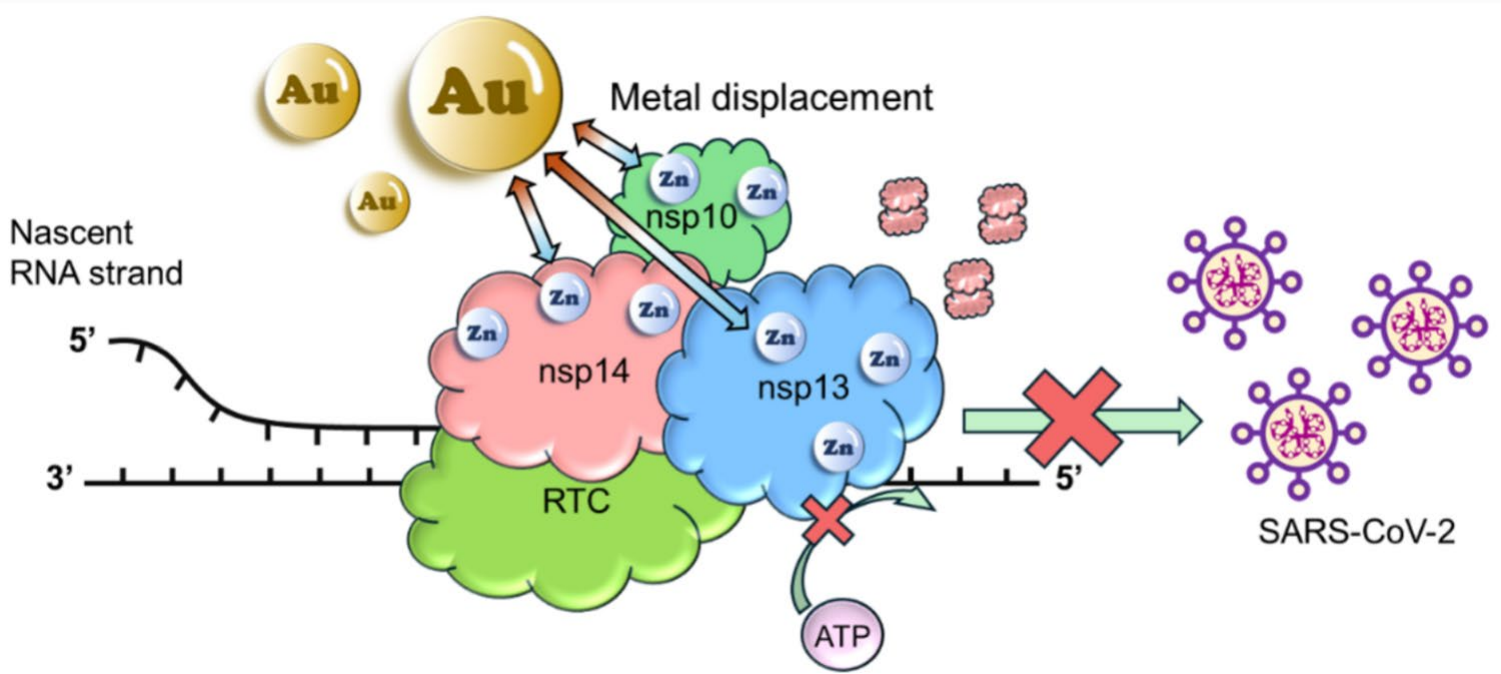

**Supplementary Information:**

The online version contains supplementary material available at 10.1007/s00775-025-02118-9.

## Introduction

The COVID-19 disease, caused by severe acute respiratory syndrome coronavirus 2 (SARS-CoV-2), has posed huge health challenges and economic loss worldwide. Although effective vaccines have been made to provide prophylactic protection for healthy adults, a large population with compromised immune systems or underlying medical conditions remains unvaccinated and the emergence of viral variants as well as immune escape mutation in the spike (S) protein also reduces vaccine effectiveness. Thus, developing potent broad-spectrum antivirals against SARS-CoV-2 and its variants is an imperative safeguard against future outbreaks and pandemics.

Upon cellular entry, SARS-CoV-2 genome encodes 16 nonstructural proteins (nsps), playing vital roles during viral transcription and replication in the host [1]. Some of these proteins have been shown to serve as druggable targets. For example, nsp3 and nsp5, which are papain-like protease (PLpro) and 3-chymotrypsin-like protease (3CLpro or Mpro) respectively, are the two mostly investigated anti-viral targets for SARS-CoV-2 and over 50 non-covalent and covalent inhibitors including GRL0617 analogues, have been reported [2]. Besides, 3CLpro inhibitors including Lopinavir, Ritonavir, Danoprevir, PF07321332 in combination with Ritonavir (Paxlovid®), ASC09F in combination with Oseltamivir, Darunavir in combination with Cobiscistat, and the noncovalent inhibitor S-217622 and Atazanavir are currently being investigated in clinical trial [3–10]. However, no rationally designed drug-like PLpro inhibitors display both in vitro and in vivo antiviral activity against both SARS-CoV-2 and MERs-CoV infection attributable to the structure differences between MERS-PLpro and SARS-PLpro. Besides, multiple mutations have been identified in Mpro among the SARS-CoV-2 and resistance to Mpro inhibitors is expected to rise with the increasing prescription [11, 12]. In addition, as Ritonavir is a potent inhibitor of the CYP3A4 isoenzyme, target selectivity and drug − drug interactions should also be taken into consideration [13]. As such, broad-spectrum antiviral strategies targeting the conserved elements of the virus replication cycle are needed to combat emerging variants and drug-resistant viruses. We anticipate that nsp14/nsp10 complex and nsp13 (helicase) stand out as two very promising targets for this purpose.

SARS-CoV-2 nsp14 functions both as an exoribonuclease together with its critical cofactor nsp10 and as a S-adenosyl methionine-dependent (guanine-N7) methyltransferase (MTase) [14]. The N-terminal ExoN domain of nsp14 plays a critical excision role in improving RNA synthesis fidelity to prevent lethal mutagenesis by removing mismatched nucleotides or nucleotide analogs from the virus ssRNA in a 3’ to 5’ direction [15]. The C-terminal domain of nsp14 displays N7-guanine MTase activity to assemble the cap structure at the 5′ end of the virus mRNA [16]. In SARS-CoV-2 nsp14/nsp10 complex, nsp10 is a critical cofactor of nsp14 which facilitates the formation of the ExoN active site, thus significantly stimulating the ExoN activity of nsp14 [17]. SARS-CoV-2 nsp13 plays an important role in helicase (nucleoside triphosphate enzymes (NTPs)) in viral replication. Nsp13 is an RNA 5’-triphosphatase and the hydrolysis of NTPs provides energy for its RNA or DNA duplexes unwinding activity with 5’-3’ directionality [18]. Sequence analysis shows that both nsp14/nsp10 complex and nsp13 are highly conserved among closely related coronavirus species, i.e., SAR-CoV-1 and MERS-CoV (Fig. S1). It is more noteworthy that nsp14/nsp10 complex and nsp13 contain five (three in nsp14 and two in nsp10) and three highly conserved zinc-binding sites, all of which play indispensable roles in enzyme activity [19, 20]. Therefore, combined with their critical functions in the viral life cycle, targeting these zinc-binding sites of nsp14/nsp10 complex and nsp13 may serve as a good strategy for the development of broad-spectrum anti-SARS-CoV-2 drugs [19, 21].

Metal-based strategy has been shown promising in combating COVID-19 [22]. Indeed, we have shown that bismuth-based drugs and compounds exhibit excellent activity against SARS-CoV-2 both in infected cells and in animal models [20, 23]. Mechanistic studies show that bismuth targets both nsp14/nsp10 and nsp13 in cells, leading to inactivating of the enzymatic activity owing to the displacement of zinc [19, 21]. In addition, a gold-based drug, auranofin, was proposed to be a promising drug against SARS-CoV-2 infection in early 2020 and soon thereafter, it was demonstrated to inhibit replication of SARS-CoV-2 in human cells at low micromolar concentration and significantly reduce the expression of cytokines induced by virus infection [24, 25]. Furthermore, oral administration of auranofin to Syrian hamsters reduced viral replication, IL-6 production, and inflammation in lungs in both therapeutic and prophylactic regimens [26]. Au(I) compounds were also shown to inhibit spike-ACE2 interaction and SARS-CoV-2 PLpro activity, thus serving as potential antivirals [27, 28]. Mechanism of Au(I) compounds against PLpro activity was also correlated to their ability to remove essential zinc ions from the enzyme [28]. As zinc fingers usually play important structural or catalytic roles in the proteins, such ejection of Zn ions from the key enzymes of SAR-CoV-2 was previously reported to be a potent strategy of clinically safe Zn-ejector drugs, disulfiram and ebselen, against virus infection [29]. Besides, it was also found that auranofin and its halido analogues as well as gold carbene complexes can inhibit SARS-CoV-2 Mpro activity, which may be related to its binding to functionally relevant cysteine residues of the protein [30]. In fact, auranofin was one of the first metallodrugs to show potential antiviral activity. A patient with human immunodeficiency virus (HIV) infection and disabling psoriatic arthritis showed improved CD4 counts and drastically reduced opportunistic infections upon auranofin treatment [31]. Following that, more and more antiviral studies were conducted on gold metallodrugs against other viral diseases including Chikungunya virus (CHIKV) and the flavivirus Zika virus [32]. Given that Au(I), a soft metal prefers to bind to enzymes containing accessible and functionally important thiol groups, zinc-binding sites of nsp14/nsp10 complex and nsp13 make them potential targets of Au(I) metallodrugs.

Herein, we showed that Au(I) metallodrugs exhibited effective anti-SARS-CoV-2 activity against both BA.5.2 and XBB strains in infected mammalian cells. Based on enzymatic studies, we found that Au(I) metallodrugs could significantly inhibit both the ExoN and MTase activities of nsp14/nsp10 complex and the ATPase and DNA unwinding activities of nsp13. Further mechanistic studies reveal that Au(I) metallodrugs could exert their anti-SARS-CoV-2 activity through displacing Zn(II) from the zinc-finger sites of nsp14/nsp10 complex and nsp13, leading to protein conformational change of nsp14 and disruption of ATP energy support for DNA unwinding of nsp13. This study highlights the potential of Au(I) metallodrugs as pan-anti-SARS-CoV-2 agents by targeting nsp14/nsp10 complex and nsp13.

## Results and discussion

### Au(I) metallodrugs inhibit SARS-CoV-2 and its variants in infected mammalian cells

Historically, Au(I) metallodrugs have been used as anti-arthritic drugs, but their antiviral activity has not been extensively explored. Previously it was shown that auranofin could inhibit the SARS-CoV-2 (USA-WA1/2020) in mammalian cells [24], which indicates the potential of Au(I) metallodrugs as anti-SARS-CoV-2 agents. Given the emergence of various SARS-CoV-2 variants, which has developed resistance to many drugs that were effective against early strains, it remains elusive whether Au(I)-based drugs/compounds serve as pan-SARS-CoV-2 agents.

**Fig. 1:**
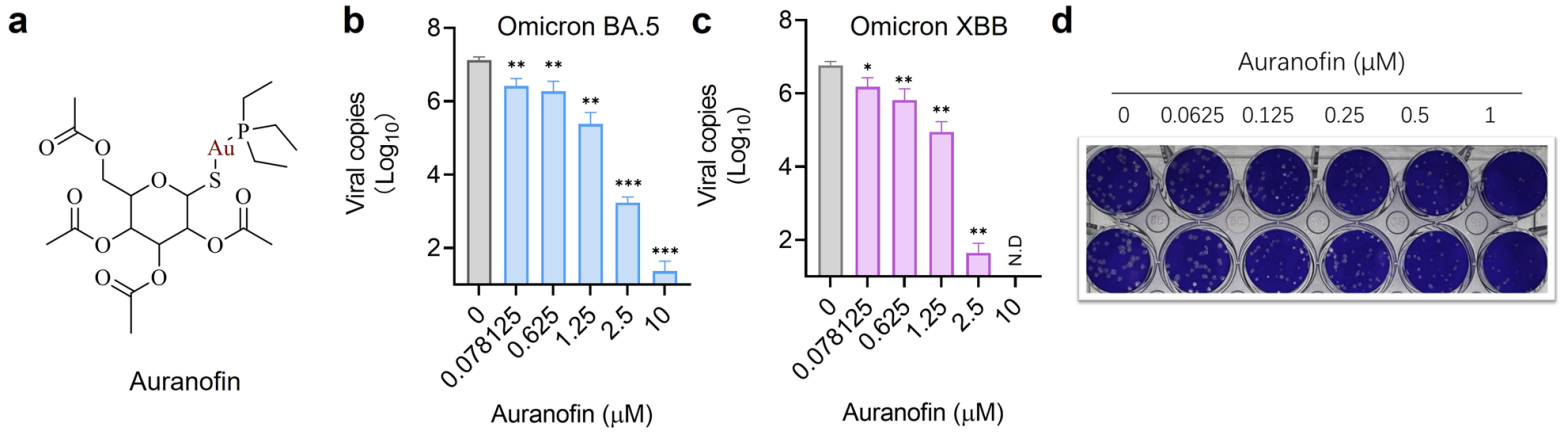
Pan-anti-SARS-CoV-2 activity of Au(I) metallodrugs in mammalian cells. **a** Structure of auranofin. **b-c** Dose-dependent inhibition of auranofin on replication of SARS-CoV-2 BA 5.2 strain (**b**) and XBB (**c**) in VeroE6-TMPRSS2 cells (MOI) = 0.01). Viral load in the cell culture supernatant was quantified by qRT-PCR at 48 h.p.i. (**h**our-**p**ost-**i**nfection). **d** EC_50_ of auranofin against SARS-CoV-2 determined by plaque reduction assay (*n* = 2). For the compounds dissolved in DMSO, the final concentration of DMSO was kept at 1%. Data are shown as mean ± SD. Statistical significance between the drug treatment group and the vehicle group (0 µM) was determined by an unpaired two-tailed Student’s *t*-test (*n* = 3); *P < 0.05, **P < 0.01 and ***P < 0.001

Since auranofin is the only Au(I) metallodrug reported so far to inhibit SARS-CoV-2 infection at the cellular level to date, we chose auranofin as a showcase to examine the pan-anti-SARS-CoV-2 activity of Au(I) metallodrugs. The pan-anti-SARS-CoV-2 potency of Au(I) was evaluated by determining its ability to inhibit viral replication in virus-infected mammalian cells. Firstly, VeroE6-TMPRSS2 cells were infected with SARS-CoV-2 variants with stronger immune escape ability (as Omicron BA.5.2 and XBB), followed by treatment with auranofin at nontoxic concentrations (0–10 μM) for 48 h [20]. Then, the viral load in the cell culture supernatant was assessed by quantitative reverse transcription polymerase chain reaction (RT-qPCR). As shown in Fig. 1, auranofin effectively reduced viral RNA loads in a dose-dependent manner, with the viral load in the cell culture supernatant reduced by a maximum of 4 − 6 log_10_ units, demonstrating its good performance in inhibiting these SARS-CoV-2 variants. A plaque reduction assay revealed that auranofin inhibited SARS-CoV-2 virus replication with a half maximal effective concentration (EC_50_) of 0.73 ± 0.21 μM, indicative of auranofin can exert pan-anti-SARS-CoV-2 activity of in mammalian cells.

### Au(I) compounds inhibit SARS-CoV-2 nsp14/nsp10 and nsp13 activity in vitro

Due to the thiophilic property of Au(I), the cysteine-rich zinc finger-containing nsp14/nsp10 complex and nsp13 are very likely to be important targets of Au(I) metallodrugs against SARS-CoV-2. To evaluate whether Au(I)-based compounds could inhibit enzymatic activity of nsp14/nsp10 and nsp13, we synthesized 14 Au(I)-based compounds and selected 5 commercially available Au(I) drugs/compounds (i.e., auranofin, Au(PEt_3_)Cl, gold(I) chloride (AuCl), gold(I) sodium thiosulfate (Na_3_Au(S_2_O_3_)_2_) and sodium aurothiomalate (AuTM)) for preliminary screening against SARS-CoV-2 nsp14/nsp10 and nsp13.

**Fig. 2:**
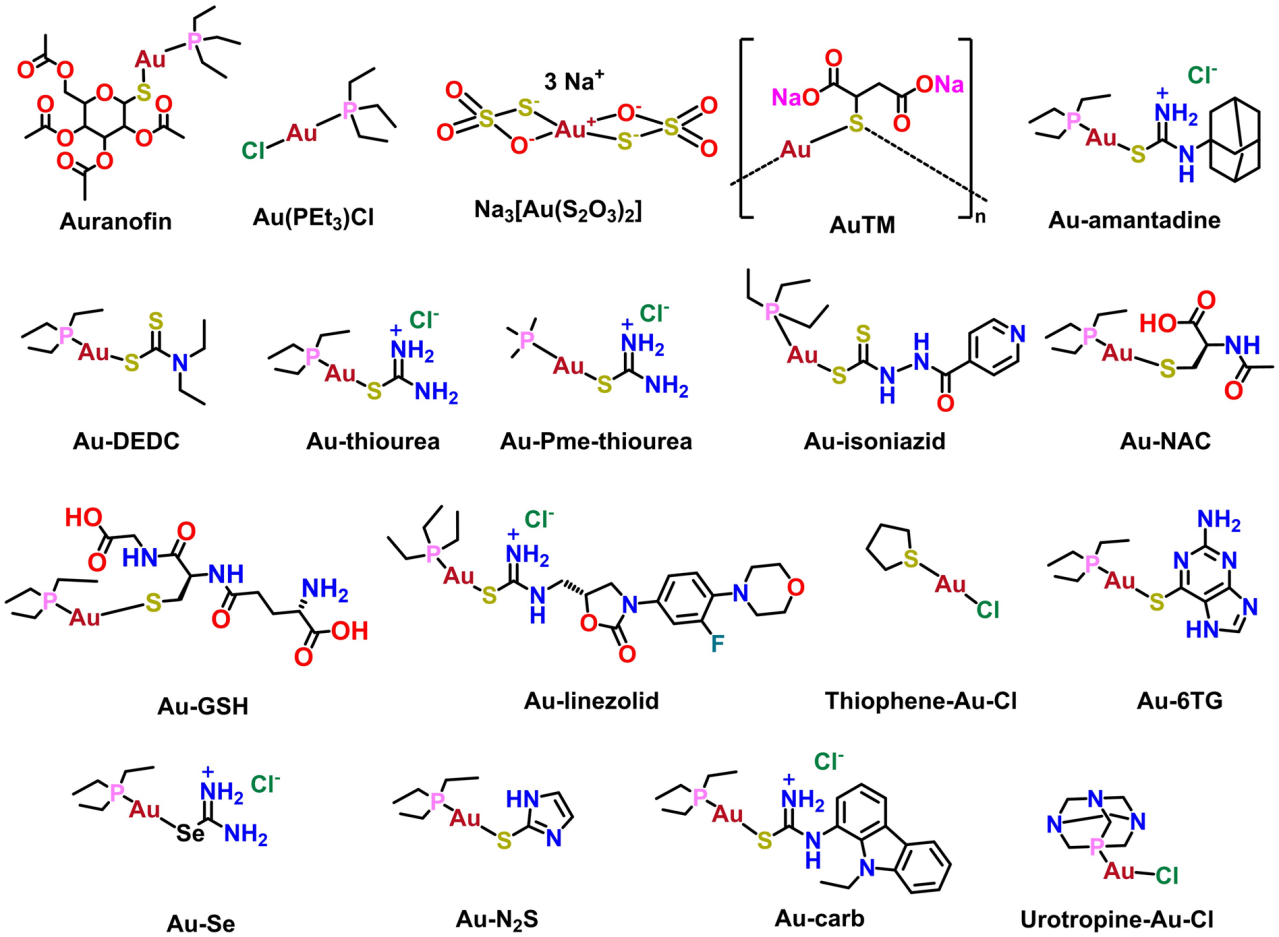
Chemical structures of selected Au(I)-based drugs/compounds

Among these compounds, auranofin, an anti-rheumatoid arthritis drug consists of three parts, phosphine, Au(I) and thiolate, in which the top phosphine part and the lower thiolate part can be modified to improve its antiviral activity. For the triethylphosphine moiety, a total of 4 groups were tested including triethylphosphine, trimethylphosphine, urotopine-phosphine and thiophene, and for the bottom thiolate’s moieties, different binding motifs such as dithiocarbamate, thiolates and thiourea were tested. These thiourea and selenourea were used which normally give much better solubility as the complexes would become positively charged and have enough stability. Chemical structures of selected compounds are shown in Fig. 2 and detailed synthesis information is shown in supporting information (SI).

For preliminary screening, firstly, we overexpressed and purified nsp10, nsp14 and nsp13 as previously reported [19–21]. We then examined the potential of Au(I)-based compounds/drugs as inhibitors of nsp14/nsp10 and nsp13 by enzymatic activity assays, colloidal bismuth subcitrate (CBS), a previously reported inhibitor was used as a positive control [19, 20]. For nsp14, the ExoN activity of its complex with nsp10 was detected by a FRET-based exoribonuclease assay, whereas MTase activity was examined by a MTase-Glo methyltransferase assay which monitors the transfer of methyl from SAM to an analog of GpppA-RNA cap structure. For nsp13, ATPase activity was examined by a typical phosphate release assay, in which the phosphate release due to ATP hydrolysis is presented as the relative percentage to evaluate ATPase activity, and its DNA unwinding activity was detected by a FRET-based DNA-duplex unwinding assay [21].

**Fig. 3:**
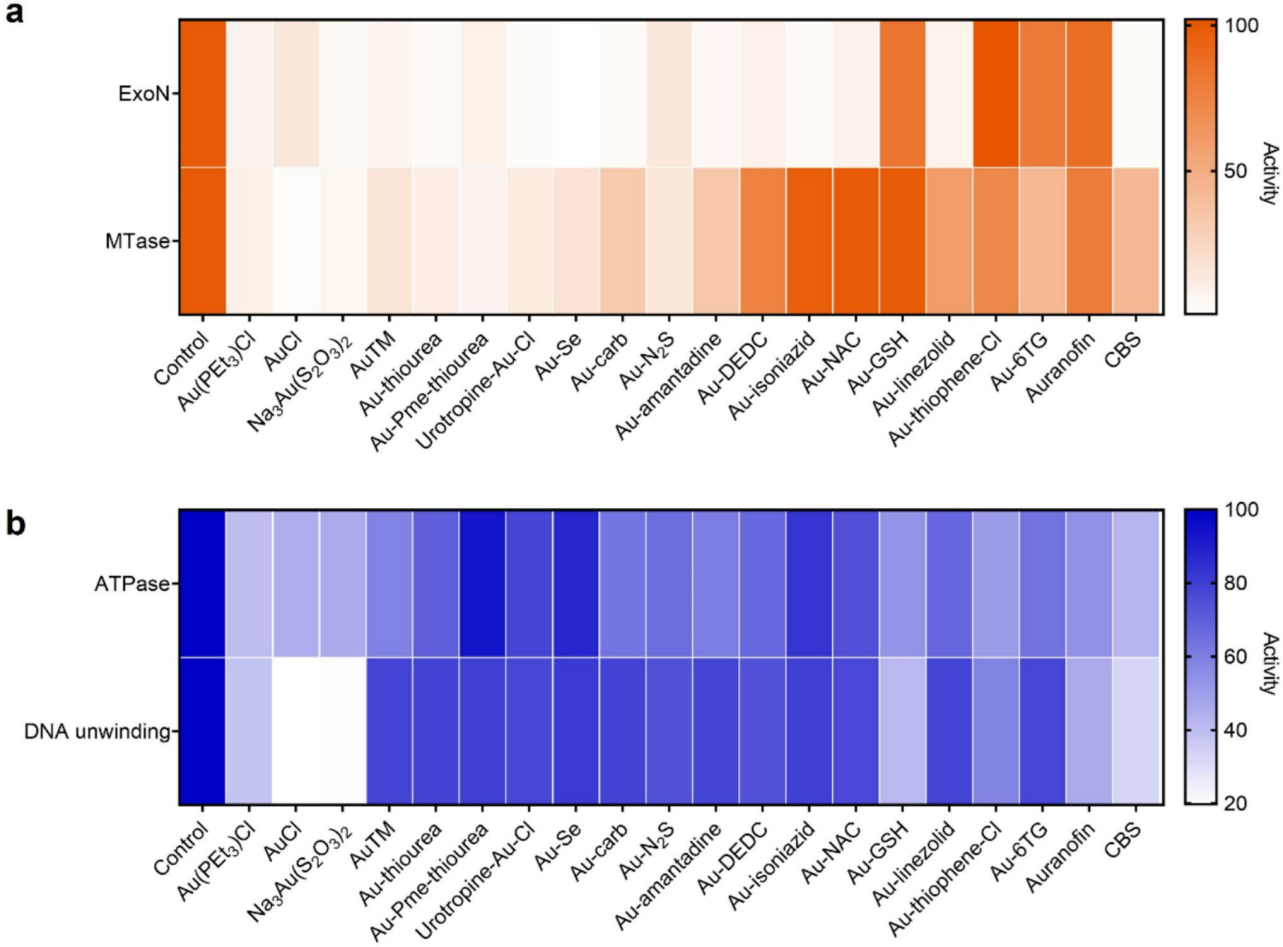
SARS-CoV-2 nsp14/nsp10 and nsp13 as potential targets of Au(I)-based compounds. ExoN and MTase activities of nsp14 (**a**) as well as ATPase and DNA unwinding activities of nsp13 (**b**) in the presence of 50 molar equivalents of Au(I)-based compounds (*n* = 3)

As shown in Fig. 3, the activities of both nsp14 and nsp13 were inhibited to varying degrees upon treatment with 50 mol equiv of Au(I)-based compounds. For both ExoN and MTase activity of nsp14/nsp10 complex, significantly, over 75% of the Au(I)-based compounds showed > 85% inhibition on ExoN activity and over 60% of Au(I)-based compounds showed > 50% inhibition on MTase activity. It is found that the p*K*_a_ of the bottom thiolate ligands is correlated to its activity. In general, the higher the p*Ka* of the thiolates, the better the inhibition. As a result, thiourea-bearing complexes have a much better inhibition than ordinally thiolates complexes, which also have a lower toxicity. However, the best phosphine groups for both functions are very different. For ExoN activity, urotropine-Au-Cl has excellent inhibition of only 0.68% enzyme activity remaining, which might be due to the electrostatic interaction between aspartate (D211 and D415) around the zinc finger and the positive charge might contribute to strongly binding and better activity, while for MTase, PMe_3_ was found to be the best phosphine group.

For both functions of nsp13, Au(PEt_3_)Cl and other gold salts showed the best results. Among other Au(I)-based compounds, Au-GSH complex also showed good results with 51.33% and 41.67% enzymatic activity remaining for ATPase and DNA unwinding activity respectively. As the zinc finger sites are far away from both active sites, the structure–activity relationship is not obvious due to minimum destruction with most of the complexes showing moderate inhibition (60–80% activity remaining). However, some compounds (Au(PEt_3_)Cl, AuCl, Na_3_Au(S_2_O_3_)_2_, etc.) that exhibited excellent inhibition on nsp14 activities could also inhibit more than 50% of ATPase and DNA unwinding activities of nsp13 simultaneously, revealing a high potential of Au(I)-based compounds as multi-targeted anti-SARS-CoV-2 agents. Based on the inhibition of both nsp14 and nsp13 by Au(PEt_3_)Cl, AuCl, Na_3_Au(S_2_O_3_)_2_ and Au-amantadine, these 4 compounds were selected for further evaluation.

**Fig. 4:**
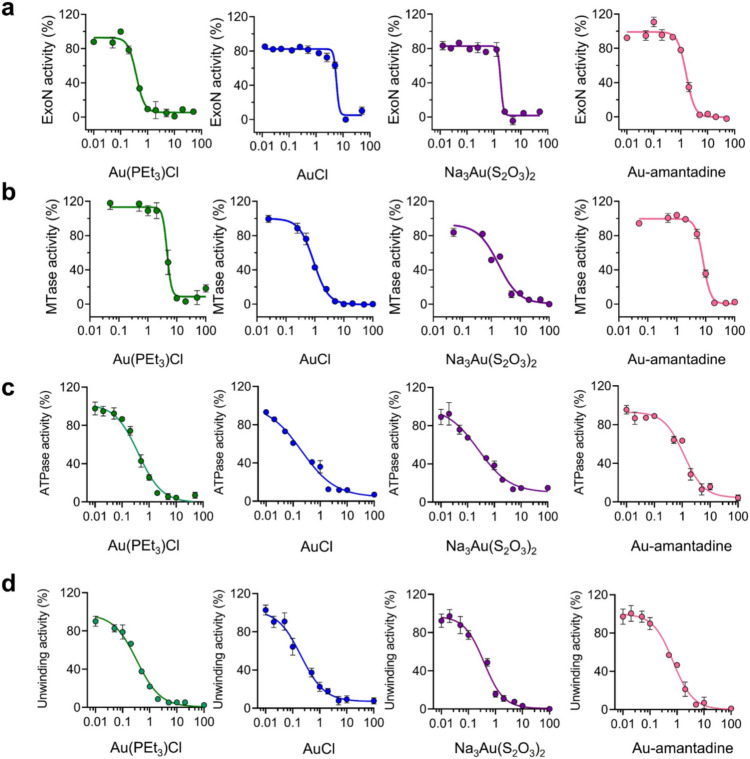
Dose-dependent inhibition of selected Au(I)-based compounds against SARS-CoV-2 nsp14/nsp10 and nsp13. ExoN (**a**) and MTase (**b**) activities of nsp14 as well as ATPase (**c**) and DNA unwinding (**d**) activities of nsp13 were examined in the presence of increasing concentrations of Au(PEt_3_)Cl, AuCl, Na_3_[Au(S_2_O_3_)_2_] and Au-amantadine (*n* = 3). IC_50_ values are presented as mean values ± SD in Table 1

As shown in Fig. 4, all these 4 Au(I)-based compounds exhibited dose-dependent inhibition on ExoN and MTase activities of nsp14 as well as ATPase and DNA unwinding activities of nsp13. Importantly, the half-maximum inhibitory concentration (IC_50_) (Table 1) of Au(PEt_3_)Cl, AuCl, Na_3_Au(S_2_O_3_)_2_ and Au-amantadine were determined to be at micromolar level, most of which are lower than 1 μM. Specifically, the IC_50_ values of Au(PEt_3_)Cl were as low as 0.38 ± 0.04 μM for nsp14 ExoN activity, and IC_50_ values of AuCl were 0.91 ± 0.01, 0.20 ± 0.01 and 0.20 ± 0.03 μM for MTase activity of nsp14, ATPase and DNA unwinding activities of nsp13, respectively, indicating the great potentials of Au(I)-based compounds as anti-SARS-CoV-2 drugs by targeting nsp14/nsp10 and nsp13.

**Table 1:** IC_50_ values for selected Au(I)-based compounds against SARS-CoV-2 nsp14/nsp10 and nsp13

Compound	IC_50_ (± SD) (μM)			
	ExoN	MTase	ATPase	DNA unwinding
Au(PEt_3_)Cl	0.38 ± 0.04	4.50 ± 0.98	0.40 ± 0.09	0.33 ± 0.04
AuCl	5.78 ± 0.31	0.91 ± 0.01	0.20 ± 0.01	0.20 ± 0.03
Na_3_Au(S_2_O_3_)_2_	1.71 ± 0.09	1.77 ± 0.01	0.25 ± 0.08	0.38 ± 0.05
Au-amantadine	1.57 ± 0.12	8.16 ± 0.18	1.15 ± 0.23	0.72 ± 0.13

### Au(I) compounds inhibit nsp14/nsp10 complex by displacing its essential Zn(II)

**Fig. 5:**
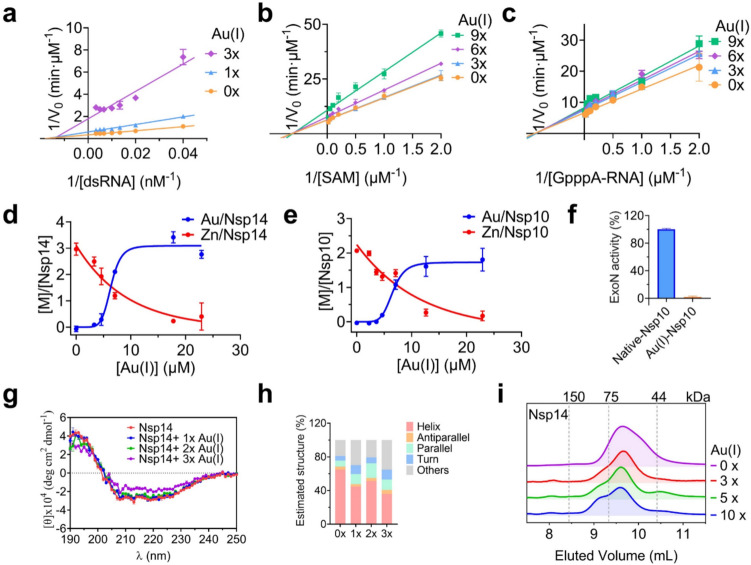
Au(I) inhibits nsp14/nsp10 complex by displacing its essential Zn(II). **a** Kinetic analysis of nsp14 ExoN activity inhibited by Au(PEt_3_)Cl. **b-c** SAM-dependent (**b**) and GpppA-RNA (**c**) kinetic analysis of nsp14 MTase activity inhibited by Au(PEt_3_)Cl. **d-e** The substitution of Zn(II) in native nsp14 (**d**) and nsp10 (**e**) by Au(PEt_3_)Cl using equilibrium dialysis. The metal content was determined by ICP-MS. **f** Relative ExoN activity of SARS-CoV-2 nsp14 in the presence of Zn-nsp10 and Au(I)-nsp10. **g-h** CD spectrum (**g**) and quantitative estimation of the secondary structure contents (**h**) of nsp14 in the presence of different molar equivalents of Au(PEt_3_)Cl. **i** Size-exclusion chromatography profiles of native-nsp14 in the presence of increasing molar equivalents of Au(PEt_3_)Cl. The elution volumes and corresponding molecular masses of the standards are shown based on the calibration using gel filtration. All data are presented as mean values ± SD (*n* = 3)

Next, we explored the inhibition mechanism of nsp14/nsp10 by Au(I). We first investigated the inhibition mode of Au(I) on the dual activities of nsp14, for which we selected Au(PEt_3_)Cl as a showcase. To explore the influence of Au(I) on the kinetics of the nsp14/nsp10 ExoN activity, a 20-nucleotide 5′-dU overhang conjugated to a Cy3/BHQ2 pair was used as an RNA duplex substrate. Then, the reciprocals of the reaction velocity and the concentration of the two substrates were used to plot the Lineweaver–Burk plot. And the Michaelis–Menten constant (*K*_m_) and the maximum reaction rate (*V*_max_) were calculated by nonlinear fitting to the Michaelis − Menten equation. We found that, in the presence of different molar equivalents of Au(I), *K*_m_ values were maintained at 40.4 ± 0.3 nM; whereas *V*_max_ values decreased from 2.7 to 0.5 nM min^−1^ (Fig. 5a), suggesting that Au(I) inhibits the ExoN activity of nsp14/nsp10 via a typical noncompetitive mode. The influence of Au(I) on the RNA-binding capability of SARS-CoV-2 nsp14 was also evaluated by an electrophoretic mobility shift assay (EMSA). Our data show that nsp14 led to a significant shift of the dsRNA substrate in the gel, confirming the formation of the nsp14 − RNA complex. Supplementation of increasing amounts of Au(I) showed negligible effects on the formation of the protein − RNA complex (Fig. S2), which is consistent with the proposed noncompetitive mode of inhibition of Au(I) on the ExoN activity of nsp14/nsp10.

In the MTase reaction of nsp14, we used S-adenosyl methionine (SAM) as the methyl donor and G(5′)ppp(5′)A RNA cap structure analog as the methyl recipient. The kinetic assays were also conducted in the presence of increasing concentrations of these two substrates, i.e., SAM and G(5′)ppp(5′)A RNA analog. Reaction progress was monitored by calculating the production of the reaction product S-adenosylhomocysteine (SAH). For the SAM-dependent kinetic assay, increasing molar equivalents of Au(I) revealed an unchanged *K*_m_ value of 1.62 ± 0.02 μM, whereas an apparent decrease in *V*_max_ values from 0.16 to 0.09 uM min^−1^ (Fig. 5b). We also observed similar trends in the G(5′)ppp(5′)A RNA-dependent kinetic assay, where the *K*_m_ values remained unchanged at 1.18 ± 0.02 μM; whereas *V*_max_ values decreased from 0.15 to 0.12 μM min^−1^ (Fig. 5c), indicative of a noncompetitive inhibition of Au(I) on MTase activity of nsp14.

Since nsp14 and nsp10 have 3 and 2 zinc-finger motifs respectively, playing structural roles and as well as being important to the functions of nsp14/nsp10 complex. The soft nature of Au(I) makes it very likely to bind to the thiolates/nitrogen atoms in these cysteine/histidine-rich zinc-binding sites. To verify this, we then investigated the binding of Au(I) to the native nsp14 and nsp10 using equilibrium dialysis and determined the metal contents by inductively coupled plasma mass spectrometry (ICP-MS). As shown in Fig. 5d, e, the supplementation of increasing amounts of Au(I) (as Au(PEt_3_)Cl) to the intact proteins resulted in *ca.* 2.95 ± 0.23 mol equiv of Au(I) bound to per nsp14 at the expense of *ca.* 2.56 ± 0.21 mol equiv of Zn(II) release. Similarly, *ca.* 1.84 ± 0.66 mol equiv of Au(I) bound to per nsp10, accompanied by *ca.* 1.89 ± 0.28 mol equiv of Zn(II) release. By fitting the data to the specific binding plot, the dissociation constants (*K*_d_) were calculated to be 6.54 ± 0.33 μM and 6.55 ± 0.77 μM and the maximal binding capacity (*B*_max_) were calculated to be 3.10 ± 0.32 and 1.75 ± 0.60 for nsp14 and nsp10 respectively, indicative of 3 and 2 Au(I) bound to nsp14 and nsp10, leading to 3 and 2 Zn(II) ions release, respectively. In addition, since the ExoN activity of nsp14 is largely stimulated by nsp10, and nsp10 − nsp14 intermolecular interaction involves residues around the zinc finger of nsp10 [33, 34], we also examined the influence of Au(I) binding to nsp10 on the ExoN activity of nsp14. In contrast to native Zn(II)-bound nsp10, Au(I)-bound nsp10 could not stimulate the ExoN activity of nsp14 (Fig. 5f), suggesting that binding of Au(I) to nsp10 might disrupt its interaction with nsp14, which further abrogates the ExoN activity of nsp14. These results suggest that Au(I)-based compounds inhibit the activity of nsp14/nsp10 through displacing the essential Zn(II) ions from the enzymes.

Metals have diversified ionic radii as well as coordination geometry and numbers, thus interaction with different metals may result in changes in protein structures and/or configurations. We then further investigated the influence of Au(I) binding on the secondary and quaternary structures of nsp14. Circular dichroism (CD) spectroscopy was used to examine the protein secondary structure changes. CD spectrum of the purified nsp14 shows two negative bands at 222 nm and 208 nm and a positive band at around 190 nm, indicating the predominant presence of α-helix structure (Fig. 5g) [35]. The spectra were then deconvoluted by *BeStSel* (Beta Structure Selection) to do the secondary structure determination and fold recognition [36], and the estimated α-helix structure content was around 65% in nsp14, as shown in Fig. 5h. Significantly, treatment of Au(I) resulted in less negative bands at 222 nm and 208 nm for the protein. Specifically, devolution of the CD spectrum revealed that after treatment with Au(I), the helix content in nsp14 was decreased to 36%, indicative of an obvious secondary structure change on nsp14 upon Au(I) binding. We also investigated the influence of Au(I) on the quaternary structure of nsp14 by size-exclusion chromatography. As shown in Fig. 5i, in the absence of Au(I), the intact nsp14 was eluted at 9.6 mL, corresponding to a molecular weight of 60 kDa (i.e. monomeric state). After incubating nsp14 with increasing molar equivalents (0 − 10) of Au(I), we observed the decreased intensities at the evolution volume of 9.6 mL (i.e. monomer peaks), and appearance and increase in the peaks at an elution volume of *ca.* 9.1 mL, suggesting that Au(I) led to partial oligomerization of nsp14. Taken together, we demonstrate that the inhibitory effect of Au(I)-based compounds on nsp14/nsp10 activity is attributable to the displacement of essential Zn(II) ion in nsp14/nsp10 by Au(I), which leads to the secondary and quaternary structure changes on the protein.

### Au(I) compounds inhibit nsp13 activity via displacement of essential Zn(II), disrupting the binding of SARS-CoV-2 nsp13 to ATP

**Fig. 6:**
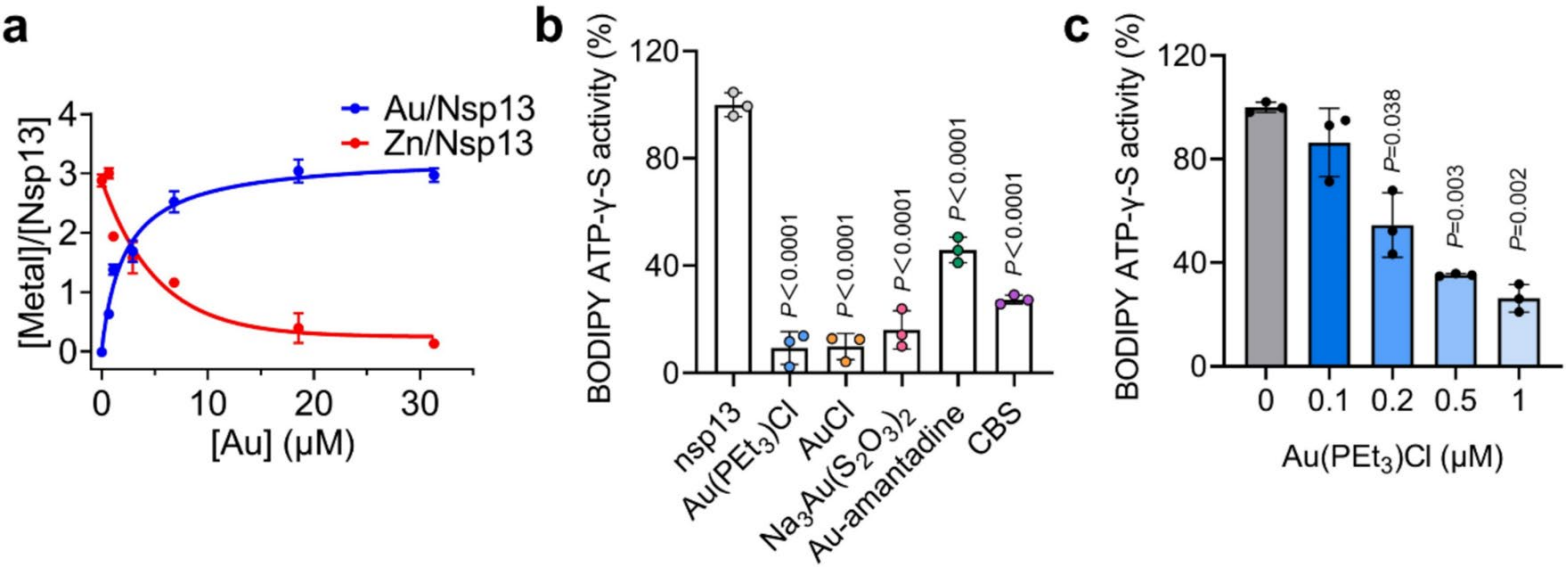
Au(I) inhibits SARS-CoV-2 helicase activities by displacing its essential Zn(II) and disrupting its binding to ATP. **a** The substitution of Zn(II) in native nsp13 by Au(PEt_3_)Cl using equilibrium dialysis. The metal content was determined by ICP-MS. **b** Fluorescence intensity of BODIPY™ FL ATP-γ-S in the presence of nsp13 with and without selected Au(I)-based compounds. **c** Dose-dependent inhibition of Au(PEt_3_)Cl in fluorescence intensity of BODIPY™ FL ATP-γ-S. All data are shown as mean ± SD. Statistical significance was calculated by an unpaired two-tailed Student's *t*-test (*n* = 3), *P < 0.05, **P < 0.01 and ***P < 0.001

Nsp13 also consists of 3 zinc finger sites at its N-terminal zinc-binding domain. Therefore, we first investigated whether Au(I) could interact with zinc fingers in nsp13 by equilibrium dialysis. Our ICP-MS results show that after the addition of increasing amount of Au(I) (as Au(PEt_3_)Cl), the native nsp13 could release *ca.* 2.88 ± 0.18 mol equiv of Zn(II) and instead bind with *ca.* 2.97 ± 0.20 mol equiv of Au(I) (Fig. 6a). By nonlinear fitting to the specific binding plot, *K*_d_ was calculated to be 2.29 ± 0.22 μM and *B*_max_ was calculated to be 3.33 ± 0.11 respectively, indicative of 3 Au(I) bound to nsp13, leading to 3 Zn(II) ions release, respectively.

To further explore the mechanism of Au(I)-based compounds against SARS-CoV-2 helicase activities, we then examined whether binding of Au(I) to nsp13 disrupt the binding of ATP to the enzyme by using adenosine 5'–O–(3–thiotriphosphate) (BODIPY™ FL ATP–γ–S), a hydrolysis resistant ATP analogue. When BODIPY ATP–γ–S binds to ATP-binding proteins, the quenched fluorescence will recover. We pre-incubated nsp13 with 1 μM selected Au(I)-based compounds (Au(PEt_3_)Cl, AuCl, Na_3_Au(S_2_O_3_)_2_ and Au-amantadine) for 10 min at room temperature, then mixed with 10 nM BODIPY™ FL ATP–γ–S, where CBS was used as a positive control. As shown in Fig. 6b, the fluorescence intensity decreased in all Au(I) treated groups, confirming the Au(I)-based compounds were able to disrupt SARS-CoV-2 nsp13 binding to ATP. Au(PEt_3_)Cl was also used as a showcase to evaluate the dose-dependent inhibitory effect of Au(I)-based compounds. The fluorescence intensity decreased in a dose-dependent manner upon treatment with 0.1, 0.2, 0.5 and 1 μM Au(PEt_3_)Cl (Fig. 6c).

Subsequently, we determined whether Au(I)-based compounds also inhibit the binding between dsDNA and protein by EMSA [21, 37]. We preincubated 2 μM nsp13 with different concentrations (0, 2, 10 and 20 μM) of Au(PEt_3_)Cl for 2 h at room temperature, then incubated with 100 nM DNA substrate to form the protein-dsDNA complex. EMSA gel image indicated Au(I) did not disrupt the binding of protein to dsDNA (Fig. S3). Collectively, we show that the Au(I)-based compound inhibits SARS-CoV-2 helicase activities by displacing the critical Zn(II) ions from its zinc-finger sites by Au(I) ions, which can disrupt the binding of protein to ATP instead of dsDNA, resulting in insufficient energy supporting unwinding activity.

## Conclusions

We have demonstrated Au(I)-based compounds as effective anti-SARS-CoV-2 agents by targeting both nsp14/nsp10 complex and nsp13. The pan-anti-SARS-CoV-2 activity of Au(I) drugs was validated in infected mammalian cell. By evaluating the ExoN and MTase activities of nsp14 as well as ATPase and DNA unwinding activities of nsp13 (helicase), we found certain Au(I)-based compounds exhibited excellent effect on inhibition of both nsp14/nsp10 and nsp13, with IC_50_ as low as less than 1 μM. Mechanistic studies showed that Au(I) compounds could inhibit nsp14/nsp10 and nsp13 activity through displacing its essential Zn(II) from the proteins by Au(I). Such metal displacement features led to secondary and quaternary structural changes of nsp14 as well as disruption of the binding of nsp13 to ATP, subsequently resulting in insufficient energy support for DNA unwinding. Taken together, our study demonstrates the importance of nsp14/nsp10 and nsp13 as the targets for Au(I)-based anti-SARS-CoV-2 agents, thus the development of effective Au(I)-based inhibitors against nsp14/nsp10 and nsp13 will be an effective strategy for combating SARS-CoV-2 and its variants due to their multi-target properties. Nevertheless, further work is required to examine the activity in vivo as well as ADME and toxicity in animals.

## Materials and methods

### Chemicals

Colloidal bismuth subcitrate (CBS) were prepared as described previously [19]. BODIPY™ FL ATP-γ-S was from Invitrogen. Certain Au(I) drugs/compounds were purchased from Sigma-Aldrich and the other 14 Au(I)-based compounds were synthesized by ourselves. All chemicals were purchased from Thermo Fisher unless otherwise stated.

### Synthesis of Au(I)-based compounds

#### General procedures for preparing substituted thiourea

Various amine (1.7 mmol) was dissolved in DCM, 1,1'-Thiocarbonyldiimidazole (TCDI) (1.9 mmol) was then added, the solution was stirred for 3 h, 20 eq. of 28% NH_3_ solution was then added and stirred overnight. NH_3_ was neutralized by 2 M HCl and 50 ml DCM was added, the organic layer was extracted with 2N HCl, the organic layer was then collected and rotary evaporated. Column chromatography (1:1 hexane: EA) was performed to give substituted thiourea.

#### General procedures for thiourea conjugate with Au(I)

Various thiourea (0.285 mmol) was dissolved in methanol, chloro(triethylphosphine)gold(I) (0.285 mmol) were added, the mixture was heated to 60 °C overnight. The solvent was then rotary evaporated and recrystallized in methanol/diethyl ether solution to yield thiourea conjugate with Au(I) complexes.

#### General procedures for thiol conjugated with Au(I)

To a solution of chloro(triethylphosphine)gold(I) (0.285 mmol) in methanol, various thiol (0.285 mmol) was added, followed by NaOMe (0.285 mmol, 1 equiv. for thiol), the solution was stirred overnight. The solvent was then rotary evaporated and redissolved in ethyl acetate, the organic layer was extracted with water or 1N HCl for carboxylate-containing ligand, the organic layer was then collected and rotary evaporated to give thiol conjugated with Au(I) complexes.

#### General procedures for dithiocarbamate conjugated with Au(I)

To a solution of chloro(triethylphosphine)gold(I) (0.285 mmol) in methanol, various dithiocarbamate sodium salt or potassium salt (0.285 mmol) was added, the solution was heated to 60 °C overnight. The solvent was then rotary evaporated and redissolved in DCM, the organic layer was extracted with water, the organic layer was then collected and rotary evaporated to give dithiocarbamate conjugated with Au(I) complexes.

#### 1-(adamantan-1-yl)thiourea (1)

Synthesized using General procedures for preparing substituted thiourea. (62.1% yield). ^1^H NMR (400 MHz, CDCl_3_): δ 5.91–6.22 (bs, 2H), δ 4.81 (s, 1H), δ 2.15 (t, 1H), δ 2.10 (d, 3H), δ 1.65–1.73 (m, 4H), δ 1.59 (s, 8H).

#### Au-amantadine (2)

(1-(adamantan-1-yl)thiourea)-(triethylphosphine)-gold(I) was synthesized using General procedures for thiourea conjugated with Au(I). (94.6% yield). ^1^H NMR (400 MHz, DMSO-d_6_): δ 1.93–2.07 (s + s + s, 10H), δ 1.87–1.92 (tt, 6H), δ 1.56–1.64 (dt, J = 12 Hz, 4 Hz, 6H), δ 1.04–1.12 (qq, 9H). ^13^C NMR (400 MHz, DMSO-d_6_): δ (ppm) 8.98, 16.73, 16.98, 28.9, 35.72, 40.04, 40.69, 52.85, 180.59. ESI–MS ( +) m/z 525.1740, found for C17H33N2PSAu [M]^+^ (calcd: 525.1762).

#### Au-DEDC (3)

((diethylcarbamothioyl)thio)-(triethylphosphine)-gold(I) was synthesized using General procedures for dithiocarbamate conjugated with Au(I) with sodium diethyldithiocarbamate trihydrate. (62.3% yield). ^1^H NMR (400 MHz, DMSO-d_6_): δ 3.79–3.80 (q, 4H), δ 1.85–1.88 (quin, 6H), δ 1.18–1.20 (m, 6H), δ 1.09–1.15 (m, 9H). The NMR data align with previous publication [38].

#### Au-thiourea (4)

(2-amino-7H-purine-6-thioate)-(triethylphosphine)-gold(I) was synthesized using General procedures for thiol conjugated with Au(I). (64% yield). ^1^H NMR (400 MHz, DMSO-d_6_): δ 12.265 (bs, 1H), δ 7.752 (bs, 1H), δ 5.934 (bs, 1H), δ 1.87–1.95 (q, 6H), δ 1.13–1.21 (tt, 9H). ^13^C NMR (400 MHz, DMSO-d_6_): δ (ppm) 8.77, 17.25, 17.47, 126.19, 137.18, 151.07, 159.22, 170.94. ESI–MS ( +) m/z 482.0841, found for C11H19N5PSAu [M + H]^+^ (calcd: 482.0843).

#### Au-Pme-thiourea (5)

((amino(iminio)methyl)thio)-(trimethylphosphine)-gold(I) was synthesized using General procedures for thiourea conjugate with Au(I). (94% yield). ^1^H NMR (400 MHz, D_2_O): δ 1.61 (s, 1H), δ 1.64 (s, 1H). ^13^C NMR (400 MHz, D_2_O): δ (ppm) 13.85, 14.10, 15.05, 15.51, 175.32. ESI–MS ( +) m/z 349.0188, found for C4H13N2PSAu^+^ [M]^+^(calcd: 349.0197).

#### Au-6TG (6)

((amino(iminio)methyl)thio)-(triethylphosphine)-gold(I) was synthesized using General procedures for thiourea conjugate with Au(I). (91.4% yield). ^1^H NMR (400 MHz, D_2_O): δ 1.04–1.12 (tt, 9H), δ 1.83–1.91 (qq, 6H). ^13^C NMR (400 MHz, D_2_O): δ (ppm) 8.3, 16.8, 17.04, 176.34. ESI–MS ( +) m/z 391.0661, found for C7H19N2PSAu^+^ [M]^+^(calcd: 391.0667).

#### 2-isonicotinoylhydrazine-1-carbodithioate (7)

Isonazid (1 g, 7.3 mmol) was dissolved in methanol was then added NaOH (0.3 g, 7.4 mmol), CS_2_ (0.427 ml, 7.4 mmol) was added dropwise, the mixture was then stirred overnight. The solvent was then rotary evaporated and redissolved in a minimum amount of methanol and then added diethyl ether, and the pale-yellow ppt. was collected. ^1^H NMR (400 MHz, DMSO-d_6_): δ 8.58–8.68 (dd, 2H), δ 7.69–7.71 (dd, 2H).

#### Au-isoniazid (8)

(2-isonicotinoylhydrazine-1-carbodithioate)-(triethylphosphine)-gold(I) was synthesized using General procedures for dithiocarbamate conjugated with Au(I). (50% yield). ^1^H NMR (400 MHz, CDCl_3_): δ 8.76 (d, 2H), δ 7.85 (d, 2H), δ 1.89–1.97 (qq, 6H), δ 1.20–1.29 (tt, 9H). ^13^C NMR (400 MHz, CDCl_3_): δ (ppm) 9.67, 17.75, 18.05, 119.31, 121.47, 140.7, 150.67, 151.09. TGA analysis: 64.5% weight loss at 750 °C in compress air, proposed structure: [Au(PEt_3_)(isoniazid-CS_2_)]_._

#### (S)-1-((3-(3-fluoro-4-morpholinophenyl)-2-oxooxazolidin-5-yl)methyl)thiourea (9)

Synthesized using General procedures for preparing substituted thiourea (57.1%). ^1^H NMR (400 MHz, DMSO-d_6_): δ 7.918 (t, 1H), δ 7.48–7.52(d, 1H), δ 7.17–7.20 (bs + d, 2H), δ 7.04–7.06 (t, 1H), δ 4.83 (t, 1H), δ 4.07–4.12 (t, 1H), δ 3.80 (s, 4H), δ 3.73 (t, 4H), δ 2.96 (t, 4H).

#### Au-linezolid (10)

(S)-(((((3-(3-fluoro-4-morpholinophenyl)-2-oxooxazolidin-5-yl)methyl)amino)(iminio)methyl)thio)-(triethylphosphine)-gold(I) was synthesized using General procedures for thiourea conjugate with Au(I) (94.5% yield). ^1^H NMR (400 MHz, DMSO-d_6_): δ 7.93 (t, 1H), δ 7.48–7.52 (d, 1H), δ 7.17–7.20 (bs + d, 2H), δ 7.04–7.06 (t, 1H), δ 4.83 (t, 1H), δ 4.07–4.12 (t, 1H), δ 3.80 (s, 2H), δ 3.73 (t, 4H), δ 3.31 (s, 2H), δ 2.96 (t, 4H), δ 1.88–1.97 (tt, 6H), δ 1.05–1.14 (qq, 9H). ^13^C NMR (400 MHz, DMSO-d_6_): δ (ppm) 8.98, 16.74, 16.99, 45.53, 46.94, 50.69, 66.14, 71.62, 106.8, 106.78, 114.16, 119.25, 119.27, 133.33, 133.4, 135.55, 135.61, 153.75, 153.99, 155.36, 183.9. ESI–MS ( +) m/z 669.1724, found for C21H34N4O3FPSAu^+^ [M]^+^(calcd: 669.1733).

#### Au-NAC (11)

(N-acetyl cysteinate)-(triethylphosphine)-gold(I) was synthesized using General procedures for thiol conjugate with Au(I) (75% yield).^1^H NMR (400 MHz, DMSO-d_6_): δ 7.88 (bs, 1H), δ 4.22–4.23 (d, J = 4 δ δ δ1.87–1.91 (tt + s, 9H), δ 1.05–1.15 (qq, 9H). The NMR align with the previous publication [39, 40].

#### Thiophene-Au-Cl (12)

Tetrachloroauric(III) acid trihydrdate (1.0 g, 2.5 mmol) was dissolved in 2 ml of water and diluted with 10 ml of ethanol. To the resultant solution, tetrahydrothiophene (0.44 mL, 5 mmol) was added dropwise, after addition the mixture was stirred at room temperature for 2 h, and then a white precipitate appeared. The precipitate was collected by filtration, washed with small amounts of ethanol and then diethyl ether to give 0.72 g (2.2 mmol) of white solid (94% yield). ^1^H NMR (400 MHz, CDCl_3_): δ 3.45 (d, 2H), δ 2.28 (d, 2H). ^13^C NMR (200 MHz, CDCl_3_): δ 40.4, 30.5. The NMR data aligns with previous publications [41].

#### Urotropine-Au-Cl (13)

Thiophene-Au-Cl (0.1 g, 0.31 mmol) was dissolved in 5 ml methanol and 1,3,5-triaza-7-phosphaadamantane (0.05 g, 0.31 mmol) was added in ice-bath. The solvent and thiophene were removed by blowing compressed air into the mixture after 3 h of reaction time. The residue was recrystallized in water/acetone mixture to give a pale-yellow cpd. (77% yield). ^1^H NMR (400 MHz, DMSO-D_6_): δ 4.33 (s, 7H), 4.36 (s, 2H), δ 4.49 (s, 2H), δ 4.51 (s, 1H). ^13^C NMR (400 MHz, DMSO-D_6_): δ 40.04, 50.82, 50.98, 71.77, 71.82. The NMR data aligns with previous publication [42].

#### Au-GSH (14)

((R)-2-((S)-4-amino-4-carboxybutanamido)-3-((carboxymethyl)amino)-3-oxopropyl)thiolate)-(triethyl-phosphine)-gold(I) was synthesized using General procedures for thiol conjugated with Au(I) (47.6% yield). Recrystallized in MeOH: diethyl ether as a white precipitate. ^1^H NMR (400 MHz, DMSO-D_6_): δ 8.17 (s, 1H), δ 4.21–4.23 (d, J = 8 Hz, 2H), δ 3.60–3.61 (d, J = 4 Hz, 2H), δ 3.33–3.37 (t, J = 8 Hz, 1H), δ 3.06 (m, 2H), δ 2.89 (m, 1H), δ 2.24–2.39 (m, 2H), δ 1.82–1.90 (m + tt, 7H), δ 1.04–1.12 (qq, 9H). The NMR data align with previous publication [40].

#### Au-N_2_S (15)

((1H-imidazol-2-yl)thiolate)gold-(triethylphosphine)-gold(I) was synthesized using General procedures for thiol conjugate with Au(I) (57.5% yield). ^1^H NMR (400 MHz, CDCl_3_): δ 6.89 (s, 2H), δ 1.85–1.93 (tt, 6H), δ 1.16–1.25 (qq, 9H). ^13^C NMR (400 MHz, CDCl_3_): δ (ppm) 9.03, 17.54, 17.77, 121.4, 146.4, 177.24. ESI–MS ( +) m/z 415.0665, found for [C9H18N2PSAu + H]^+^, [M + H]^+^(calcd: 415.0666).

#### Carbazole-thiourea (16)

1-(9-ethyl-9H-carbazol-3-yl)thiourea was synthesized using General procedures for preparing substituted thiourea (75.4%). ^1^H NMR (400 MHz, DMSO-d_6_): δ 9.63 (s, 1H), δ 8.13–8.15 (d, J = 8 Hz, 1H), δ 8.03 (s, 1H), δ 7.47–7.61 (t, J = 8 Hz, 2H), δ 7.44–7.46 (t, J = 4 Hz, 1H), δ 7.30–7.32 (d, J = 8 Hz, 1H), δ 7.17–7.20 (t, J = 8 Hz, 2H), δ 4.41–4.46 (q, J = 8 Hz, 2H), δ 1.29–1.31 (t, J = 8 Hz, 3H).

#### Au-carb (17)

1-(9-ethyl-9H-carbazol-3-yl)isothiouronium)-(triethylphosphine)-gold(I) was synthesized using General procedures for thiourea conjugate with Au(I) (89.5% yield). ^1^H NMR (400 MHz, DMSO-d_6_): δ 8.14–8.17 (d, J = 8 Hz, 1H), δ 8.04–8.06 (t, J = 8 Hz, 1H), δ 7.56–7.62 (q, J = 4 Hz, 2H), δ 7.44–7.46 (t, J = 4 Hz, 1H), δ 7.31–7.33 (d, J = 8 Hz, 1H), δ 7.19–7.22 (t, J = 4 Hz, 2H), δ 4.41–4.46 (q, J = 8 Hz, 2H), δ 1.85–1.93 (tt, 6H), δ 1.29–1.31 (t, J = 8 Hz, 3H), δ 1.03–1.08 (qq, 9H). ^13^C NMR (400 MHz, DMSO-d_6_): δ (ppm) 8.92, 13.73, 16.88, 16.92, 37.01, 109.2, 109.28, 117.2, 118.75, 126.0, 121.97, 122.22, 123.72, 125.97, 129.73, 137.65, 139.96, 179.57. ESI–MS ( +) m/z 584.1559, found for [C21H30N3PSAu]^+^, [M]^+^(calcd: 584.1558).

#### Au-Se (18)

((amino(iminio)methyl)seleno)-(triethylphosphine)-gold(I) was synthesized using General procedures for preparing substituted thiourea (75.4%). ^1^H NMR (400 MHz, D_2_O): δ 1.83–1.91 (tt, 6H), δ 1.04–1.13 (qq, 9H). ESI–MS ( +) m/z 439.0118, found for [C7H19N2PSeAu]^+^, [M]^+^(calcd: 439.0111).

### Cell lines and viruses

VeroE6-TMPRSS2 cells were cultured in Dulbecco’s Modified Eagle Medium (DMEM), 10% fetal bovine serum (FBS) and 1% antibiotics (penicillin and streptomycin) at 37 °C. All experiments involving live SARS-CoV-2 followed the approved standard operating procedures of the Biosafety Level 3 facility at the Department of Microbiology, The University of Hong Kong.

### Cellular antiviral activity assay

A viral load reduction assay was performed for the evaluation of cellular antiviral activity, as described previously but with modifications [43, 44]. Briefly, SARS-CoV-2-infected (MOI = 0.01) VeroE6-TMPRSS2 cell were treated with different concentrations of Au(I)-based compounds. The culture supernatants of the infected cell were harvested at 48 h-**p**ost-**i**nfection (h.p.i.) for analysis of viral replication. The supernatant was collected by 50 μL RLT buffer, and subsequently extracted for total RNA with the QIAamp viral RNA mini kit (Qiagen). Quantitative reverse transcription-polymerase chain reaction (qRT-PCR) was used for quantitation of SARS-CoV-2 viral load using the One Step TB Green® PrimeScript™ RT-PCR Kit II (Takara) with a LightCycler 480 Real-Time PCR System (Roche). The primers and probe sequences were against the RNA-dependent RNA polymerase/Helicase (RdRP/Hel) gene region of SARS-CoV-2: forward primer: 5'CGCATACAGTCTTRCAGGCT-3'; reverse primer: 5'-GTGTGATGTTGAWATGACATGGTC-3'; specific probe: 5'-FAMTTAAGATGTGGTGCTTGCATACGTAGAC-IABkFQ-3'.

### Plaque reduction assay

VeroE6-TMPRSS2 cells (2 × 10^5^ per well) were seeded in 24-well plate. Next day, cells were infected with 50 plaque-forming units (PFU) of SARS-CoV-2 Omicron BA.5, then treated with the compounds in a dose-dependent manner or without drugs as control. Monolayers were then overlaid with media containing 1% low melting agarose inverted and incubated for another 72 h. Then, the wells were fixed with 10% formaldehyde for 12 h. After removal of the agarose plugs, monolayers were stained with 0.7% crystal violet and plaques were counted.

### Cloning, protein expression, and purification

SARS-CoV-2 nsp10 and nsp14 were overexpressed and purified as previously described [19]. Firstly, plasmids pET28a-nsp10 and pET28a-nsp14 were generated by cloned full-length nsp10 and nsp14 of SARS-CoV-2 isolate Wuhan-Hu-1, complete genome (NCBI GenBank accession no. NC_045512.2) into pET-28-a( +) vector with a N-terminal 6 × His-tag, respectively. Next, the plasmids were transformed into *E. coli* DH10B and validated by sequencing. After validation, plasmids were then transformed into Rosetta (DE3) pLysS, respectively. Cells containing pET28a-nsp10 and pET28a-nsp14 were cultured at 37 °C overnight in 2 × YT medium supplemented with 50 μg·ml^−1^ kanamycin and 20 μg·ml^−1^ chloramphenicol, respectively. Then, cells were amplified at 37 °C in 2 × YT medium at a 100-fold dilution and when OD_600_ reached 0.7, the nsp10 and nsp14 were overexpressed by induction of 0.05 mM and 0.3 mM isopropyl β-D-thiogalactoside (IPTG) for nsp10 and nsp14, respectively. After induction for 18 h 16 °C with agitation at 160 rpm, cells were harvested by centrifugation at 5000*g* for 30 min at 4 °C, washed twice with phosphate-buffered saline (PBS), and then resuspended and lysed by sonication in bacteria lysis buffer (PBS containing 5 mM b-mercaptoethanol (only for nsp10), 0.1% Triton X-100, 1 mM phenylmethylsulfonyl fluoride (PMSF), 40 mM imidazole, and 10 mg/ml DNase I). The cell lysates were then centrifuged at 15,000 g for 45 min at 4 °C, and recombinant nsp10 and nsp14 were purified using Ni–NTA column (Invitrogen) using a gradient of imidazole-containing Tris-NaCl buffer (50 mM Tris–HCl, pH 7.5, 300 mM NaCl, 5 mM MgSO_4_) for washing and elution. Thrombin Protease was used to remove His tags from nsp10 and nsp14. Further, nsp10 was purified by a Superdex 200 Increase 10/300 GL column (GE Life Sciences) with gel-filtration buffer (20 mM HEPES, pH 7.4, 150 mM NaCl, 5 mM MgCl_2_, 5% glycerol), and nsp14 was purified using HiTrap S ion-exchange chromatography (GE Healthcare). Purified nsp10 and nsp14 were examined by SDS-PAGE and the identity of the proteins was further confirmed by LC–MS after trypsin digestion. Finally, proteins were aliquoted and stored in HEPES buffer (20 mM HEPES, pH 7.4, 150 mM NaCl, 5 mM MgCl_2_ and 5% glycerol) at -80 °C for further use.

SARS-CoV-2 helicase was overexpressed and purified as previously described [20, 21]. Briefly, *E. coli* BL-21(DE3) cells harboring pET-28a-nsp13 plasmid were cultured in LB medium with 50 μg/mL kanamycin overnight and then diluted by 1:100 to 4 L fresh LB medium. Cells were cultured to OD_600_ of 0.6–0.8, then induced by 200 μM IPTG at 16 °C for 18 h with agitation at 200 rpm. The bacteria were collected at 6000 g, 4 °C for 30 min. Cell pellets were resuspended in lysis buffer (20 mM Tris, 500 mM NaCl, pH 7.4, 0.1% Triton X-100) with Protease Inhibitor Cocktail and sonicated. The cell lysate was centrifuged (12,000*g*, 4 °C for 10 min), proteins was purified by Ni–NTA Agarose (Invitrogen). SARS-CoV-2 helicase was eluted by 250 mM imidazole and checked by SDS-PAGE. The purest fractions were removed His_6_-tag by thrombin. The protein concentrations were quantified by a bicinchoninic acid (BCA) assay kit.

### Methyltransferase (MTase) activity assay

A MTase-Glo™ Methyltransferase Assay Kit (Promega) was used to measure the MTase activity and a G(5’)ppp(5’)A RNA Cap Structure Analog (New England Biolabs) was used as the substrate for the assay. Progression of the reactions was monitored by luminescence in white 384-well plates (Corning) using a SpectraMax iD3 multimode microplate reader.

For primary screening, before the methyltransferase reaction, nsp14 (5 µM) was pre-incubated with Au(I) drugs (250 µM) at room temperature for 1 h. Next, 8 µL reactions were carried out by incubating 1 µM nsp14 treated with a serial dilution of drugs, 20 µM for SAM and 20 µM for G(5’)ppp(5’)A RNA Cap Structure Analog in MTase reaction buffer (20 mM Tris–HCl, pH 8.0, 50 mM NaCl, 3 mM MgCl_2_, 0.1 mg/mL BSA, 0.5 mM TCEP) at room temperature for 30 min. Then, a 2 µL 5 × Methyltransferase-Glo Reagent was added and the reactions were incubated at room temperature for 30 min. After that, 10 µL of Methyltransferase-Glo Detection Solution were added, followed by incubation for another 30 min at room temperature. The progress of the reactions was then monitored by luminescence using a microplate reader.

For determination of the half-maximum inhibitory concentration (IC_50_), firstly, nsp14 (10 µM) was pre-incubated with Au(I) drugs (0, 0.5, 5, 10, 20, 50, 100, 200, 500, 1000 µM) at room temperature for 1 h before the methyltransferase reaction. Next, MTase reactions were carried out and detected as described in primary screening. Luminescence was then monitored by a microplate reader and dose-dependent inhibition curves were generated and IC_50_ value were calculated by nonlinear regression using GraphPad Prism.

For the MTase kinetic assays, an SAH standard curve was first generated to assess the linearity of the assay and to calculate the amount of the reaction product SAH produced in each reaction. 8 µL of MTase reaction buffer containing serial diluted SAH (from 0 to 1 µM) were incubated with 2 µL of 5 × Methyltransferase-Glo Reagent for 30 min at room temperature followed by the addition of 10 µL of detection solution as described above. Luminescence was recorded and then plotted against SAH concentration by linear regression using GraphPad Prism. For methyltransferase kinetic reaction, nsp14 (5 µM) was pre-incubated with Au(I) drugs (as Au(PEt_3_)Cl) at the molar ratio (as indicated in the data) at room temperature for 1 h. MTase kinetic assays were carried out in 8 µL of MTase reaction buffer containing drug-treated nsp14 (1 µM), 2 µL 5 × Methyltransferase-Glo Reagent, together with 25 µM G(5’)ppp(5’)A RNA Cap Structure Analog and serially diluted SAM (0, 0.5, 1, 2, 5, 10, 20 and 50 µM) for SAM-dependent kinetic assays, or 25 µM SAM and serially diluted G(5’)ppp(5’)A RNA Cap Structure Analog (0, 0.5, 1, 2, 5, 10, 20 and 50 µM) for G(5’)ppp(5’)A RNA-dependent kinetic assays at room temperature for 10 min, followed by adding 2 µL 0.5% trifluoroacetic acid (TFA) to stop the reaction. After that, 10 µL of Methyltransferase-Glo Detection Solution were added and mixed well, followed by incubation for another 30 min at room temperature. The control group was performed in the absence of drugs under the same condition. The amounts of SAH produced in different intervals were calculated by the standard curve to determine the velocity of the reaction. The velocity and the concentration of the substrates were used to generate the Lineweaver–Burk plot. *K*_m_ and *V*_max_ were calculated by the Michaelis–Menten equation using GraphPad Prism.

### FRET-based exoribonuclease (ExoN) activity assay

A slightly modified double-stranded RNA with a 20-nucleotide 5’ dU overhang, conjugated to a Cy3-quencher (BHQ2) pair, was used as the substrate for the exoribonuclease activity assay based on a previous report (see Table S1) [45]. Nsp14 and nsp10 were mixed at a molar ratio of 1:4 to form the nsp14-nsp10 complex. Progression of the reactions was monitored by fluorescence (λ_ex_ = 545 nm, λ_em_ = 585 nm) in black 96-well plates (Thermo Fisher Scientific) using a SpectraMax iD3 multimode microplate reader. ExoN activities were calculated by the amount of fluorescence released after subtracting the initial reading.

For screening the inhibitor, 5 µM of nsp14-nsp10 complex were pre-incubated with 250 µM drugs at room temperature for 1 h. Then, the reaction was carried out in 20 µL of ExoN reaction buffer (50 mM Tris–HCl, pH 8.0, 30 mM NaCl, 2.5 mM MgCl_2_, 10 mM K_2_HPO_4_, 0.5 mM tris(2-carboxyethylphosphine (TCEP) and 0.1 mg/mL BSA) containing 200 nM nsp14/nsp10 complex, 10 µM drugs, and 50 nM Cy3/quencher-dsRNA substrates at 25 °C for 2 h. The fluorescence signal was monitored by the SpectraMax iD3 multimode microplate reader in real time.

For the half-maximum inhibitory concentration (IC_50_) calculation, nsp14-nsp10 complex (10 µM) was pre-incubated with Au(I) drugs (0, 0.25, 1.25, 2.5, 5, 12.5, 25, 50, 125, 250, 500 and 1250 µM) at room temperature for 1 h before the nuclease reaction. Nuclease assays (20 µL) were performed at 25 °C for 2 h in ExoN reaction buffer in the presence of 50 nM Cy3/quencher-dsRNA substrates and 200 nM drug-pretreated nsp14/nsp10 complex. Dose–response curves for IC_50_ value were determined by nonlinear regression using GraphPad Prism.

For the ExoN kinetic assays, nsp14-nsp10 complex (10 µM) was incubated with an equal volume of drugs at the specified molar ratio for 2 h on ice before the nuclease reaction. Nuclease kinetic assays were carried out in 100 µL ExoN reaction buffer containing 100 nM (for RBC) and 50 nM (for Au(PEt_3_)Cl) nsp14-nsp10 complex incubated with drugs, and serially diluted Cy3/quencher-dsRNA substrates (0, 25, 50, 75, 100, 150, 200, 300, 400 and 500 nM). The control group was performed in the absence of drugs under the same condition. The maximum of the first derivative over the first hour was calculated to determine the velocity of the reaction. The velocity was plotted against the concentration of the substrates. Slopes were then used to calculate the K_m_ and V_max_ by nonlinear fitting to the Michaelis–Menten equation using GraphPad Prism.

### ATPase assay

ATPase inhibitory activities of gold-based compounds against SARS-CoV-2 helicase were assayed using a commercial assay kit (ab234055, Abcam). The experiments were performed following the manufacturer’s instruction. In brief, we pre-incubated 20 nM nsp13 with different drugs/ concentrations drugs in a 50 μL ATPase reaction buffer for 10 min at room temperature. Next, the other 50 μL ATPase reaction buffer which included 1 µL substrate was added into the reaction system for 20 min. Finally, 15 µL developer was added to the well to react with the released phosphate. After 30 min incubation, the absorbance was measured by SpectraMax iD3 Multi-Mode microplate reader at 650 nm. The IC_50_ values were determined by nonlinear regression using GraphPad Prism. The assays were performed in triplicate.

### FRET DNA duplex unwinding assay

The FRET-based DNA unwinding activity assay was performed as the previously described method [21, 46]. DNA oligos were purchased from Integrated DNA Technologies (see Table S1). The two oligomers mixed at a ratio of FL-Cy3: RL-BHQ of 1:1.5 for the final concentrations of 10 μM and 15 μM, respectively were annealed in annealing buffer containing 20 mM Tris–HCl pH 8.0, 150 mM NaCl by heating to 90 °C for 2 min in thermocycler (S1000™ Thermal Cycler, Bio-Rad), then cooling slowly to 25 °C at the rate of ~ 1 °C/min. 20 nM protein was pre-incubated with different metallodrug / concentrations in 95 μL reaction buffer (20 mM Tris–HCl buffer, pH 7.4, 150 mM NaCl, 5 mM MgCl_2_, 5 mM TCEP, 0.1 mg/ml BSA and 10% glycerol) in a 96-well black polystyrene microplate for 10 min at 25 ℃. Then, 2 μL 100 mM ATP, oligomers (FL-Cy3, RL-BHQ mixture) were added to result in the final concentration of FL-Cy3: RL-BHQ oligo and RL oligo at 5 nM and 50 nM respectively. Fluorescence intensity was measured at excitation/emission wavelengths 550/620 nm by SpectraMax iD3 Multi-Mode microplate reader. IC_50_ values were determined by nonlinear regression using GraphPad Prism. The assays were performed in triplicate.

### Electrophoretic mobility shift assay (EMSA)

RNA-binding capability of nsp14 was determined by EMSA. For the protein dose-dependent RNA-binding assays of nsp14, 200 nM of the ExoN substrate dsRNA were incubated with varying concentration of SARS-CoV-2 nsp14 (0, 0.4, 0.8, 1.2, 1.6, 2.0, 4.0, 8.0 µM) in HEPES buffer at room temperature for 15 min. For the bismuth(III) and Au(I) addition assays, nsp14 (4 µM) was pre-incubated with various concentrations of drugs (0, 1, 2, 4, 8, 20, 40 µM) in HEPES buffer for 2 h on ice before the RNA-binding assays before incubating with 200 nM of dsRNA at room temperature for 15 min. A 20 µL of the reaction mixture was then loaded onto 6% native-polyacrylamide gels and electrophoresed in 1xTBE buffer. Electrophoresis was carried out at 100 V for 50 min. Gels were subsequently stained by GelRed (Sigma Aldrich) for 5 min at room temperature and visualized through iBright™ CL750 Imaging System (Thermo Fisher Scientific).

EMSA for investigating the binding between nsp13 and DNA substrates was performed as previously reported [19]. Briefly, 2 μM nsp13 was incubated with Au(PEt_3_)Cl in a dose-dependent manner in 20 μL binding buffer (20 mM Tris pH 7.4, 20 mM NaCl, 1 mM TCEP, 5 mM MgCl_2_) for 2 h at 25 °C. Then, incubated per sample at 37 °C with 100 nM dsDNA. After 15 min incubation, the reaction was stopped by 4 μL loading buffer (50% glycerol, 0.02% bromophenol blue). The reaction mixture was electrophoresed in 8% native-polyacrylamide gels at 150 V for 1 h. The gel was stained by GelRed (Sigma Aldrich) for 10 min and images were captured by iBright™ CL750 Imaging System (Thermo Fisher Scientific).

### Metal displacement analysis

Au(I) binding and Zn(II) release on nsp10 and nsp14 were determined by Inductively Coupled Plasma Mass Spectrometry (ICP-MS) on Agilent 7700 × ICP-MS (Agilent Technologies). SARS-CoV-2 nsp14, nsp13 and nsp10 were incubated with 3 molar equivalents of ZnSO_4_ in dialysis buffer (20 mM HEPES, pH 7.4, 150 mM NaCl, 5 mM MgCl_2_, 5% glycerol, 0.5 mM TCEP) with mild shaking at 4 °C overnight, followed by removal of unbound zinc(II) in Zn-free dialysis buffer to make the fully Zn-bound proteins. Dialysis tubes loaded with 100 μL Zn-bound proteins (5 μM for nsp14 and nsp13 and 20 μM for nsp10) or protein buffer were placed into 50 mL dialysis buffer in the presence of different concentrations of Au(I) compounds (as Au(PEt_3_)Cl) with mild shaking at 18 °C overnight. The protein concentration was measured by bicinchoninic acid (BCA) assay, and the metal concentration of solution both inside and outside the dialysis tubes was determined by ICP-MS.

### Circular dichroism (CD) spectroscopy

CD spectra were collected by a Jasco J-815 Spectrometer. Nsp14 (3 μM) was incubated with indicated molar equivalents of drugs (as Au(PEt_3_)Cl) for 1 h on ice in 10 mM Na_2_HPO_4_ buffer (pH 7.0), respectively. CD Spectra were detected between 190 to 250 nm at room temperature in a 0.1 cm quartz cuvette with a scanning rate of 50 nm/min. Each spectrum was scanned for three times and then subjected to the online CD spectrum analysis server *BeStSel* [36].

### Size-exclusion chromatography analysis

Size-exclusion chromatography was performed with a Superdex 75 increase 100/200 GL analytical column (Cytiva) at 4 °C. The column was pre-equilibrated by PBS buffer. 20 μM nsp10 or 5 μM nsp14 were pre-incubated with different molar equivalents of Au(PEt_3_)Cl for 1 h at 4 °C. Samples were prepared in PBS buffer to a final volume of 500 μL before loading to the column at a flow rate of 0.8 mL/min.

### ATP-γ-S fluorescence assay

Pre-incubated 100 nM nsp13 with different drugs / concentrations of drugs in 50 μL reaction buffer (20 mM This, 500 mM NaCl, pH 7.4, 5 mM MgCl_2_) for 10 min at room temperature. Then, 10 nM BODIPY™ FL ATP-γ-S was added. Fluorescence signals were measured by SpectraMax iD3 Multi-Mode microplate reader at excitation/emission wavelengths 502/545 nm. The assays were performed in triplicate. Data are shown as mean ± SD. The statistical significance was calculated by unpaired two-tailed Student's *t*-test (n = 3), *P < 0.05, **P < 0.01, and ***P < 0.001.

### Statistical analysis

All statistical analyses were performed on three independent experiments, or more if otherwise stated, using Prism 8.0 (GraphPad Software Inc.).

## Supplementary Information

Below is the link to the electronic supplementary material.

Supplementary file1 (PDF 2916 KB)

Supplementary file2 (PDF 563 KB)

## Data Availability

No datasets were generated or analysed during the current study.
