# Supplementary material for: Au(I)-based compounds inhibit nsp14/nsp10 and nsp13 (helicase) to exert anti-SARS-CoV-2 properties
Source: J Biol Inorg Chem. 2025 Jun 18;30(4-5):425–41. doi: 10.1007/s00775-025-02118-9 (PMC12316777; doi:10.1007/s00775-025-02118-9)

# Supporting Information

# Au(I)-based compounds inhibit nsp14/nsp10 and nsp13 (helicase) to exert anti-SARS-CoV-2 properties

Jingxin Chen,[‡1,2] Xueying Wei,[‡1,2] Chun-Lung Chan,[1] Kaiming Tang,[2] Shuofeng Yuan,[2] Hongyan Li,[1] and Hongzhe Sun*[1]

[1] Department of Chemistry and HKU-CAS Joint Laboratory of Metallomics on Health and Environment, The University of Hong Kong, Pokfulam Road, Hong Kong SAR, China.

[2] Department of Microbiology and State Key Laboratory of Emerging Infectious Diseases, The University of Hong Kong, Pokfulam Road, Hong Kong SAR, China.

Correspondence and requests for materials should be addressed to H.S. (email: hsun@hku.hk).

[‡] These authors contribute equally to this work.

## Supplementary Figures

## Supplementary Table

## Compound Synthesis Spectra and TGA Analysis

# Supplementary Figures

## a. Nsp14

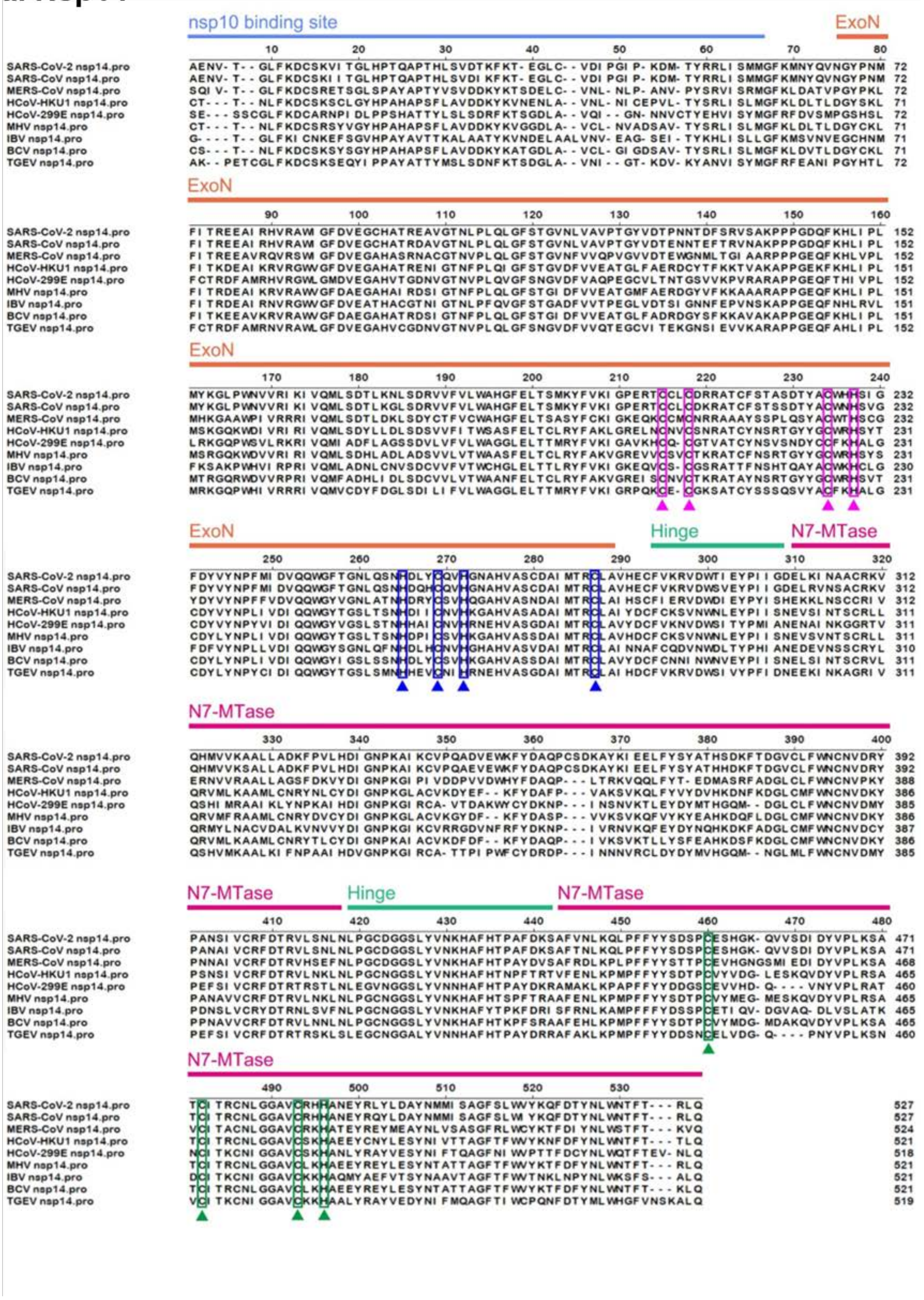

## b. Helicase

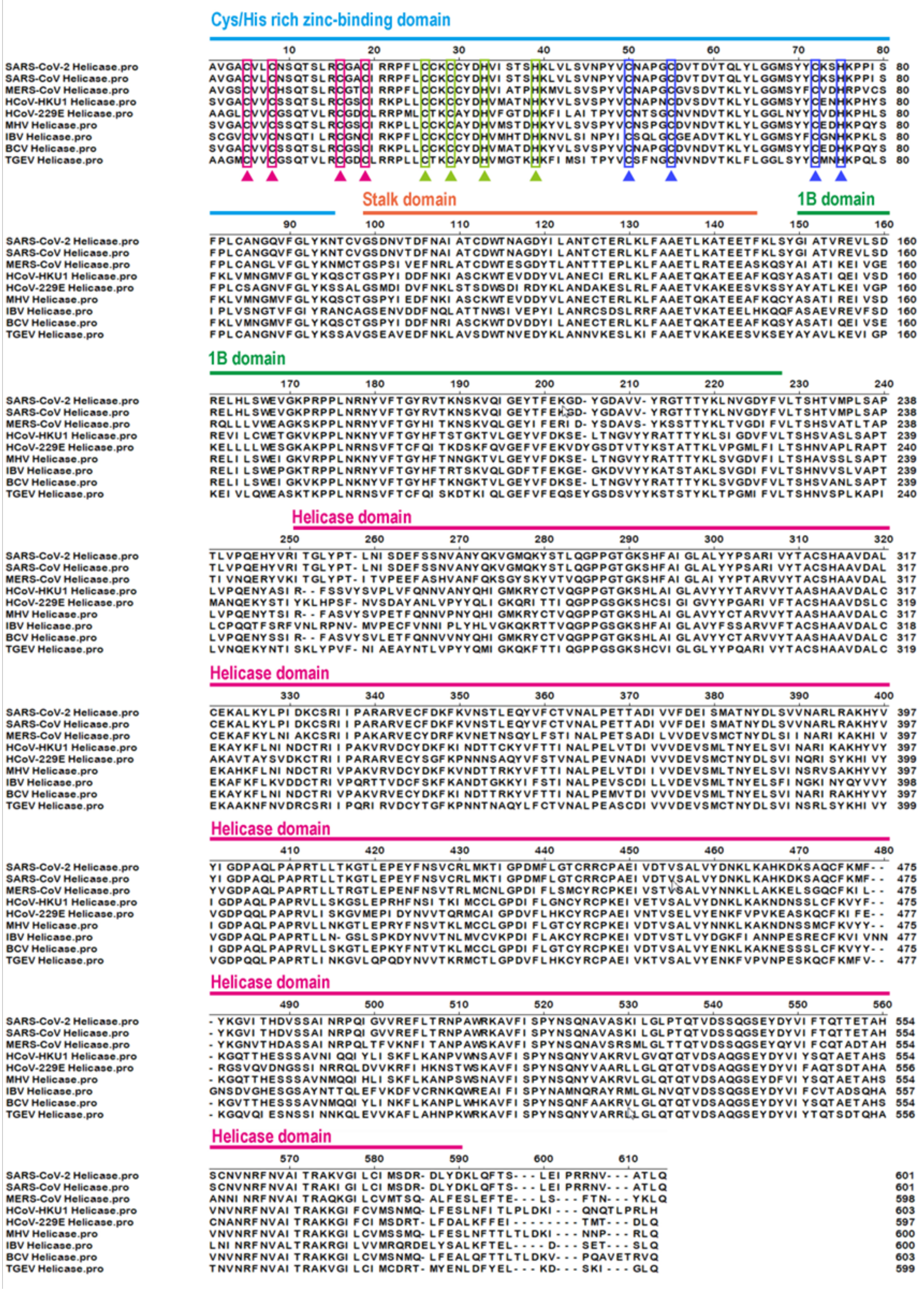
Cys/His rich zinc-binding domain
Stalk domain
1B domain
Helicase domain
SARS-CoV-2 Helicase.pro
SARS-CoV Helicase.pro
MERS-CoV Helicase.pro
HCoV-HKU1 Helicase.pro
HCoV-229E Helicase.pro
MHV Helicase.pro
IBV Helicase.pro
BCV Helicase.pro
TGEV Helicase.pro

**c. Nsp10**

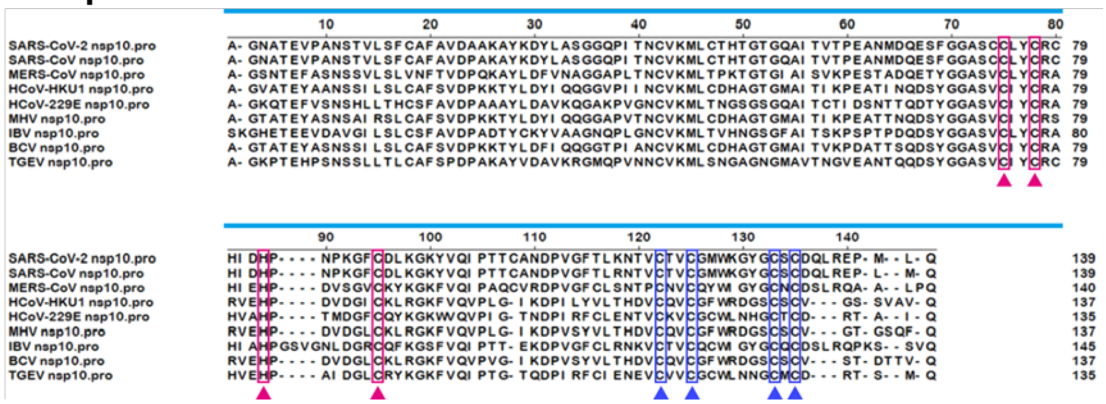


**Fig. S1** Sequence alignment of nsp14, nsp13 (helicase) and nsp10 from selected coronaviruses using MegAlign (DNASTAR) software. Sequences of nsp14 (**a**), nsp10 (**b**) and helicase (**c**) in MERS-CoV (NC-019843), SARS-CoV-2 (NC_045512.2), SARS-CoV (NC_004718.3), HCoV-HKU1 (NC_006577.2), MHV (NP_045299.2), HCoV-229E (NC_002645.1), IBV (NC_001451.1), BCV (NP_150073.3), TGEV (AJ271965.2), and were used for the analysis. Residues involved in the formation of three zinc fingers are marked with pink, blue and green triangles.

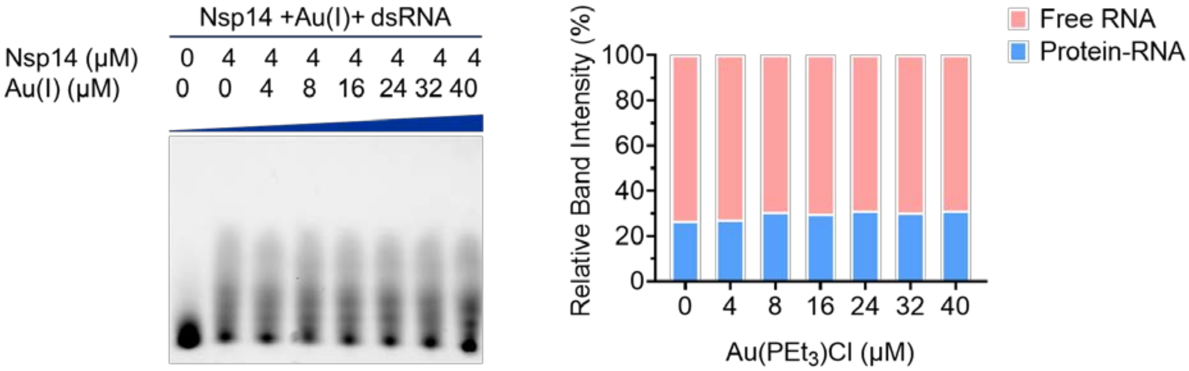


**Fig. S2** Influence of Au(I)-based compounds (as Au($PEt_3$)Cl) on the binding of SARS-CoV-2 nsp14 to ExoN substrates. Relative band intensities were quantified by ImageJ.

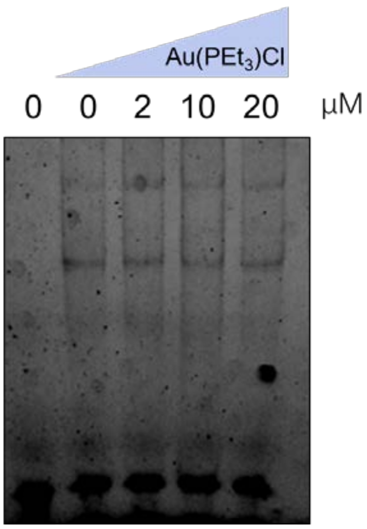


**Fig. S3** EMSA image of the binding between DNA substrate and nsp13 in the presence of increasing amounts of Au(I)-based compound (as $Au(PEt_3)Cl$).

## Supplementary Table

**Table S1 Sequence of oligonucleotides.**

| No. | Oligo | Oligo sequence (5'→3') |
|---|---|---|
| i | Top Cy3 RNA for ExoN activity | Cy3-GGUAGUAAUCCGCUC |
| ii | Bottom quencher RNA for ExoN activity | UUUUUUUUUUUUUUUUUUUUGAGCGGAUUACUACUACC-BHQ-2 |
| iii | FL-Cy3 oligo for DNA unwinding activity | TTTTTTTTTTTTTTTTT TTTTCGAGCACCGCTGCGGCTGCACC-Cy3 |
| iv | RL-BHQ oligo for DNA unwinding activity | BHQ2-GGTGCAGCCGCAGCGGTGCTCG |
| v | RL oligo for DNA unwinding activity | GGTGCAGCCGCAGCGGTGCTCG |

# Compound Synthesis Spectra and TGA Analysis

## 1-(adamantan-1-yl)thiourea (1)

400 MHz $^1$H NMR in $CDCl_3$

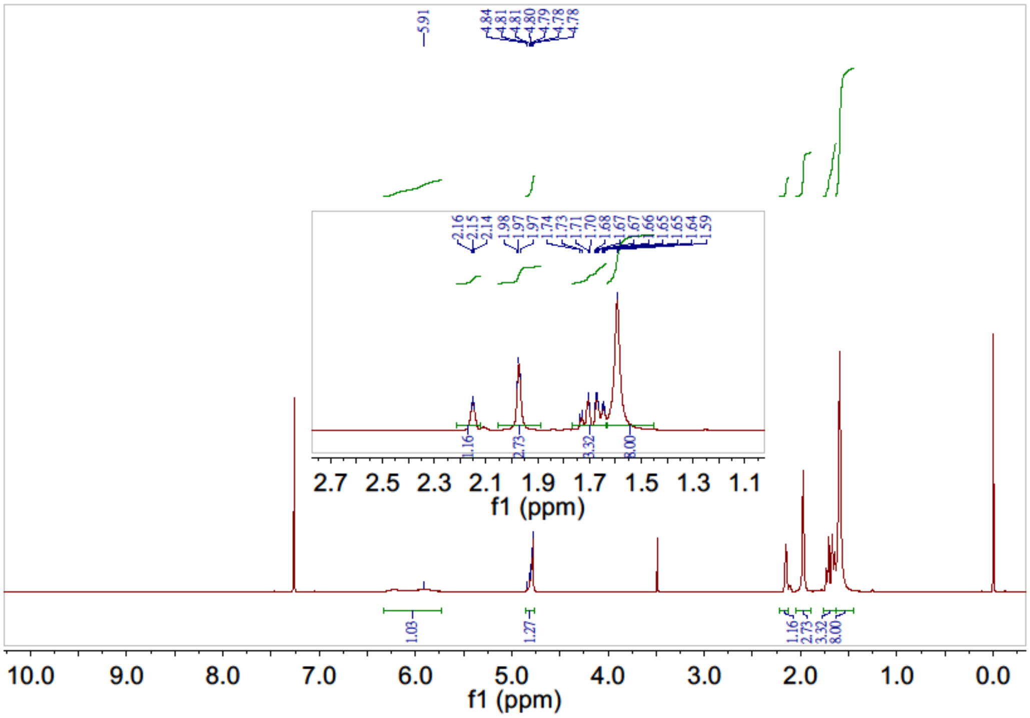

**Au-amantadine (2)**

1. 400 MHz $^{1}H$ NMR in DMSO-$d_6$

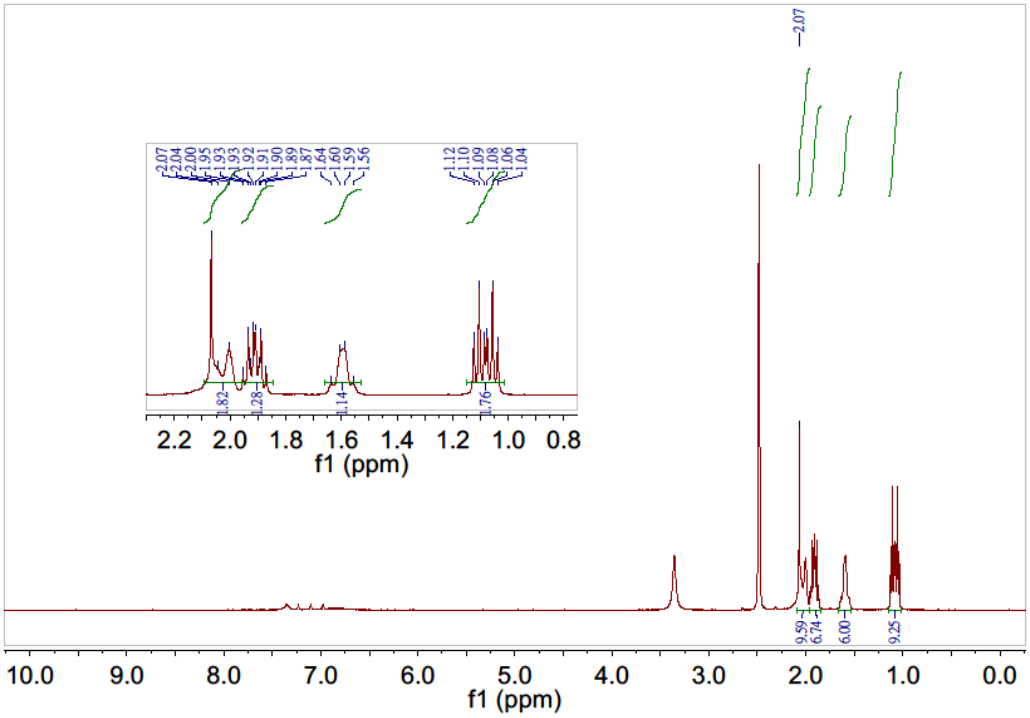

2. 400 MHz $^{13}C$ NMR in DMSO-$d_6$

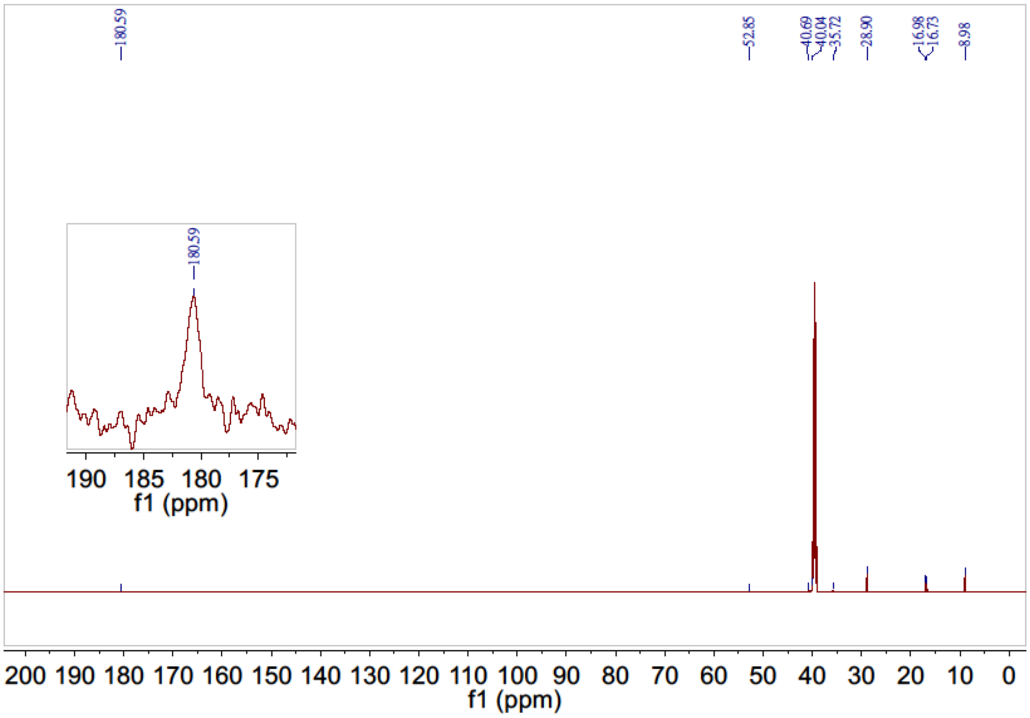

3. ESI-MS

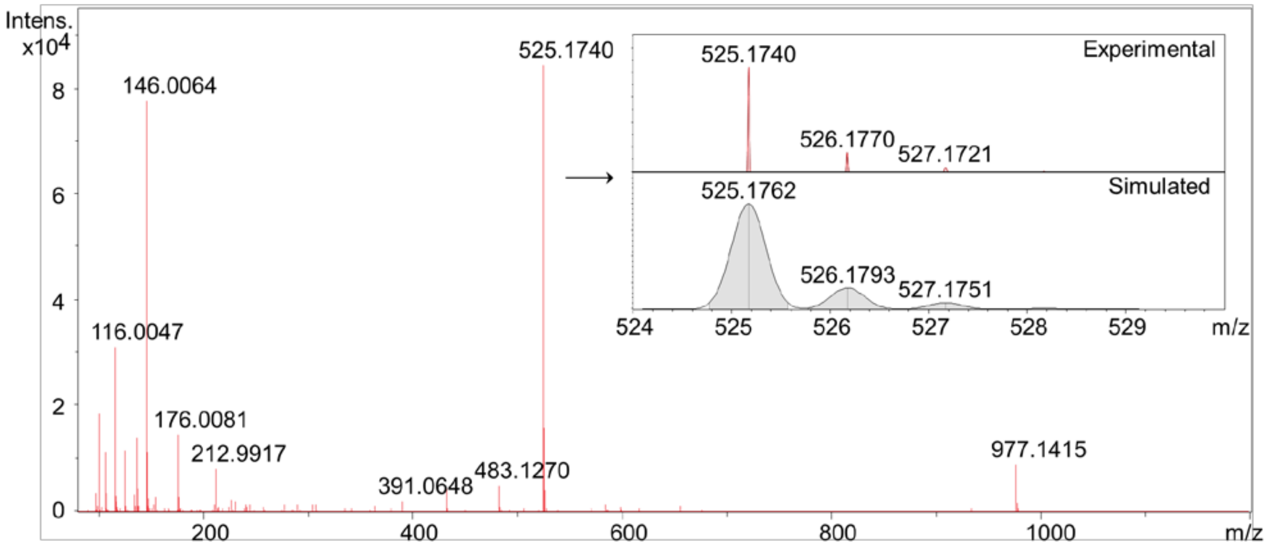

Intens.
x10^4
146.0064
116.0047
176.0081
212.9917
391.0648
483.1270
525.1740
977.1415
m/z
Experimental
526.1770
527.1721
Simulated
525.1762
526.1793
527.1751

**Au-DEDC (3)**

1. 400 MHz $^1$H NMR in DMSO-$d_6$

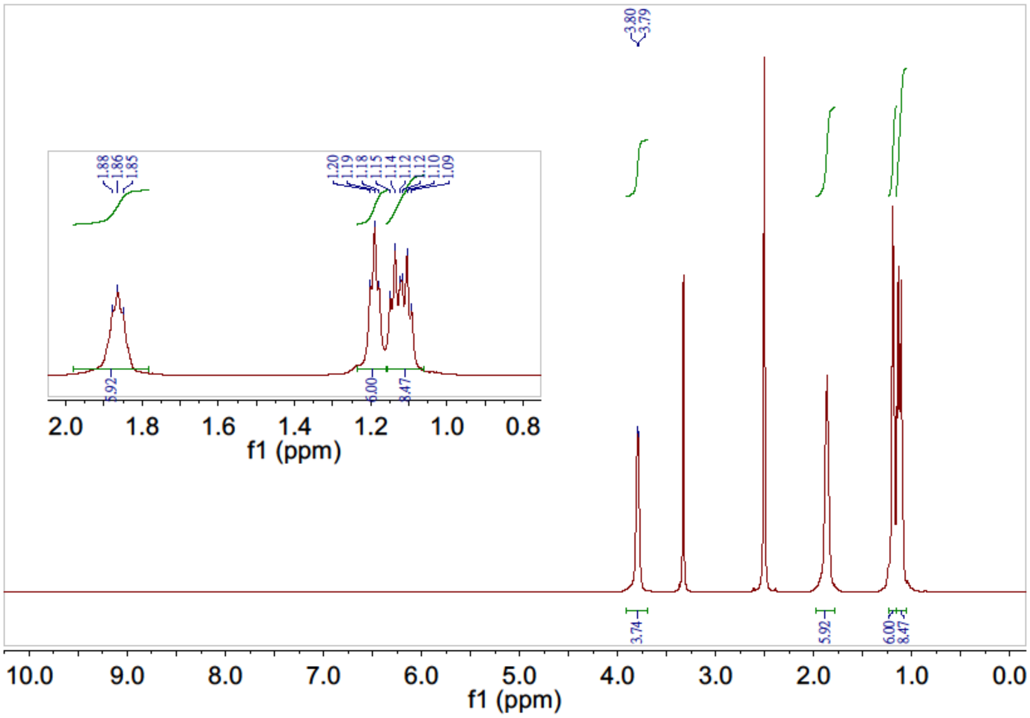

**Au-thiourea (4)**

1. 400 MHz $^{1}H$ NMR in DMSO-d6

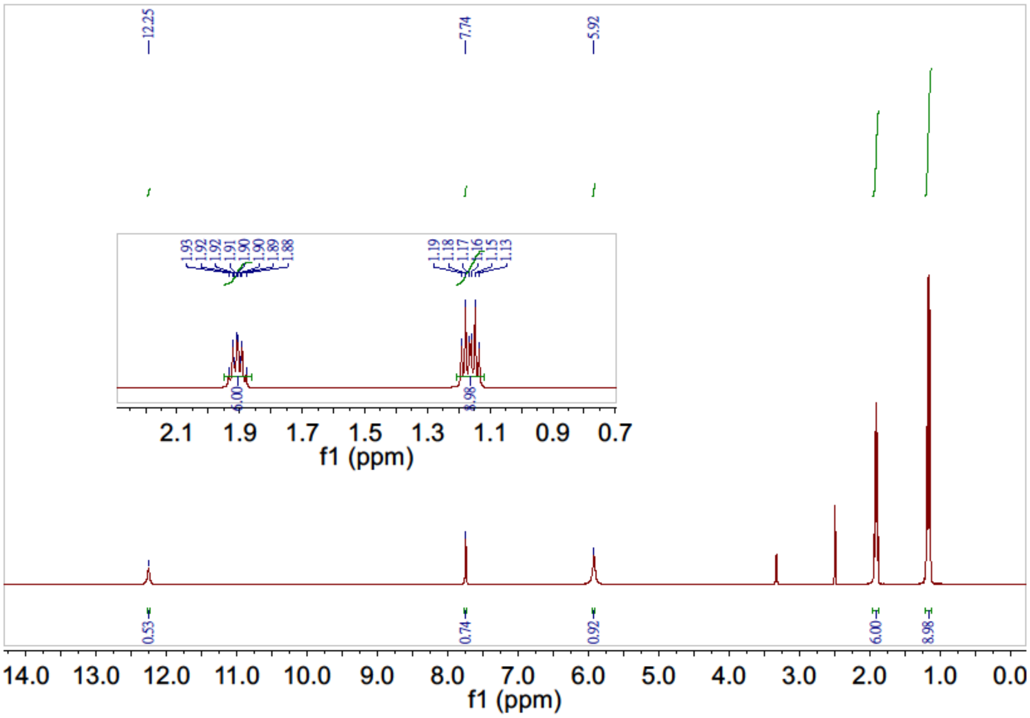


2. 400 MHz $^{13}C$ NMR in DMSO-d6

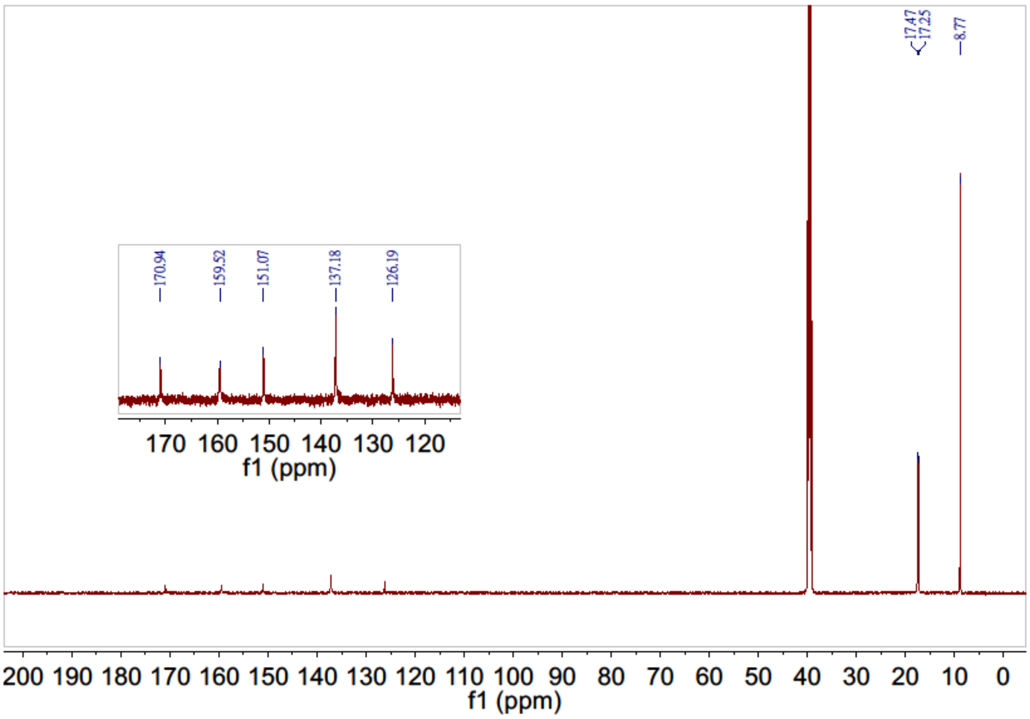

3. HR-ESI-MS

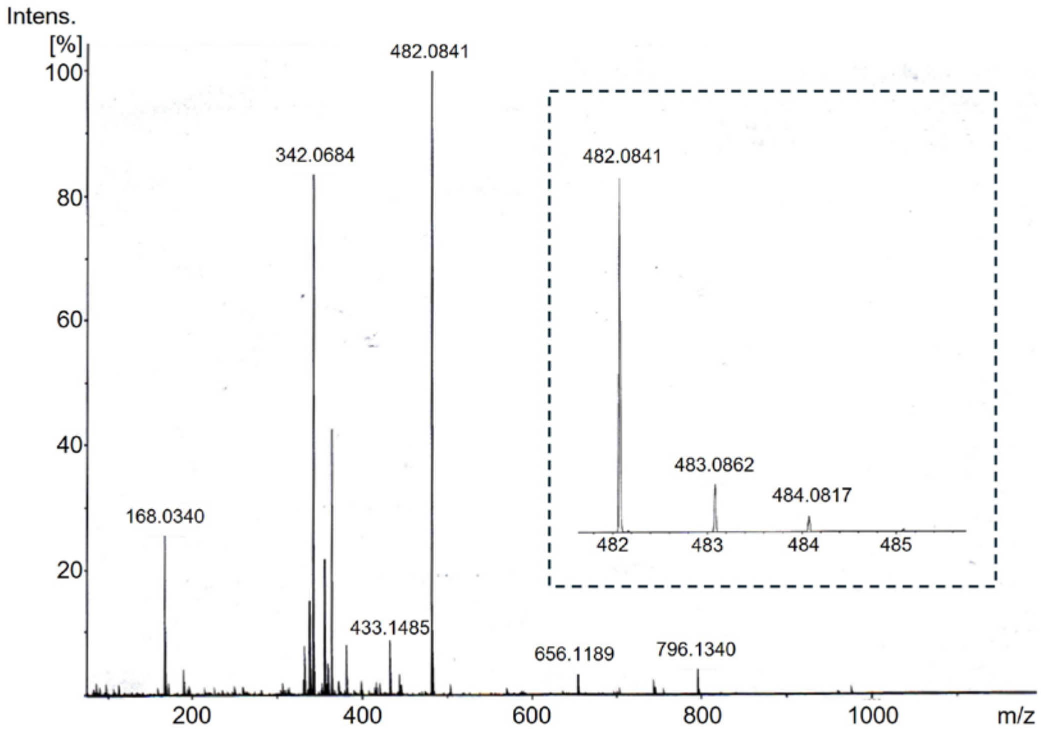
Intens.
[%]
100
80
60
40
20
482.0841
342.0684
168.0340
433.1485
656.1189
796.1340
200
400
600
800
1000
m/z
482.0841
483.0862
484.0817
482
483
484
485

**Au-Pme-thiourea (5)**

1. 400 MHz $^{1}H$ NMR in $D_2O$

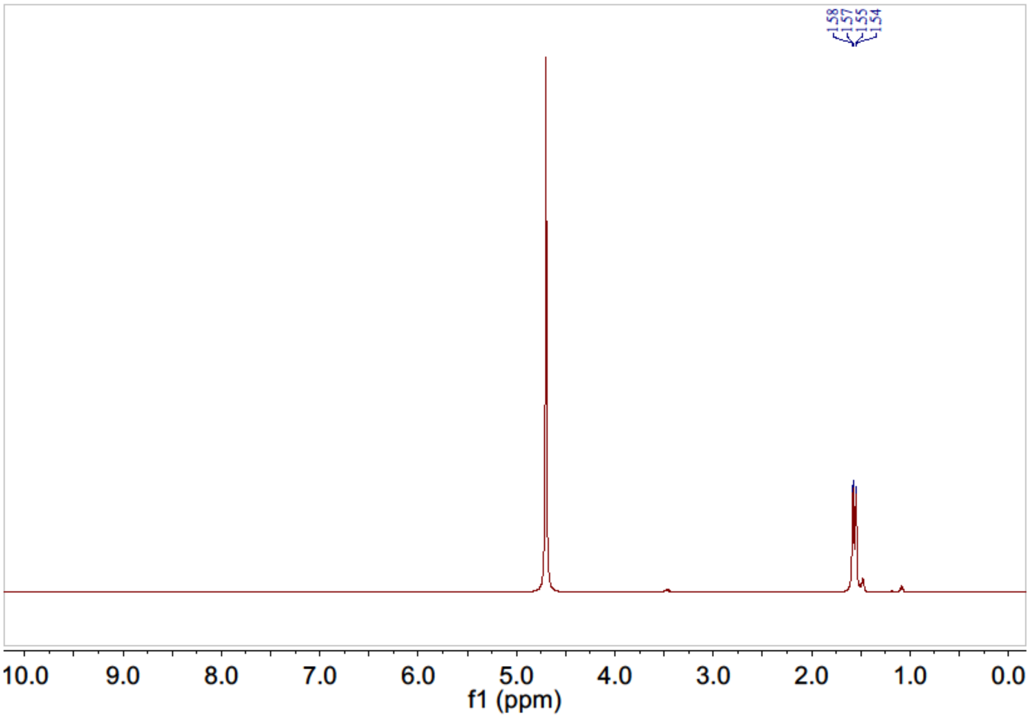


2. 400 MHz $^{13}C$ NMR in $D_2O$

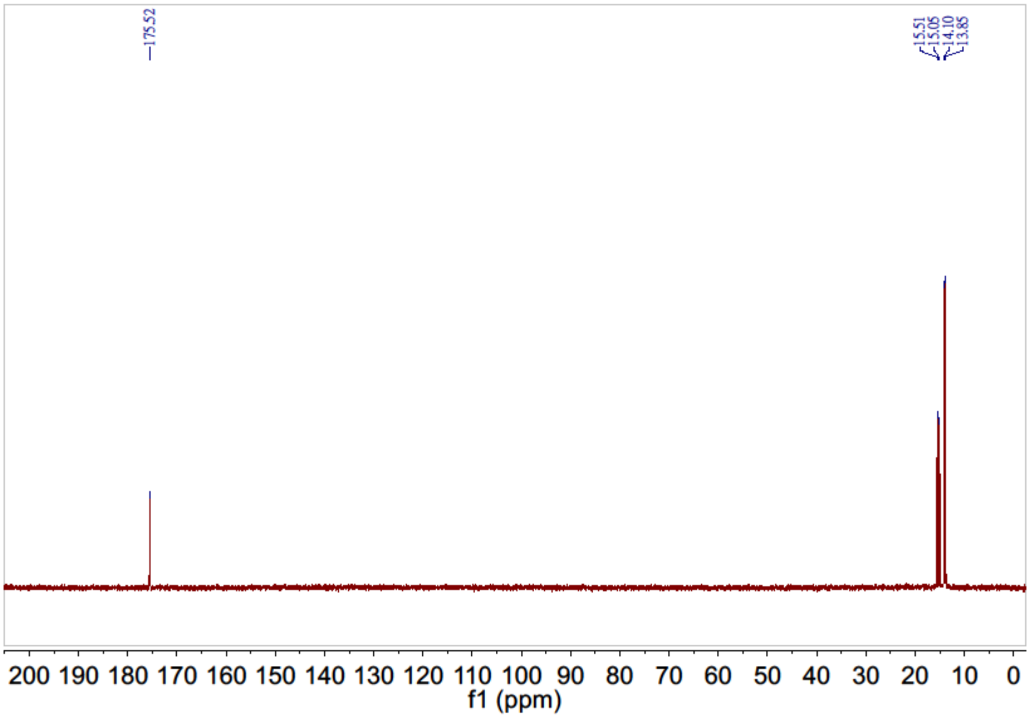

3. HR-ESI-MS

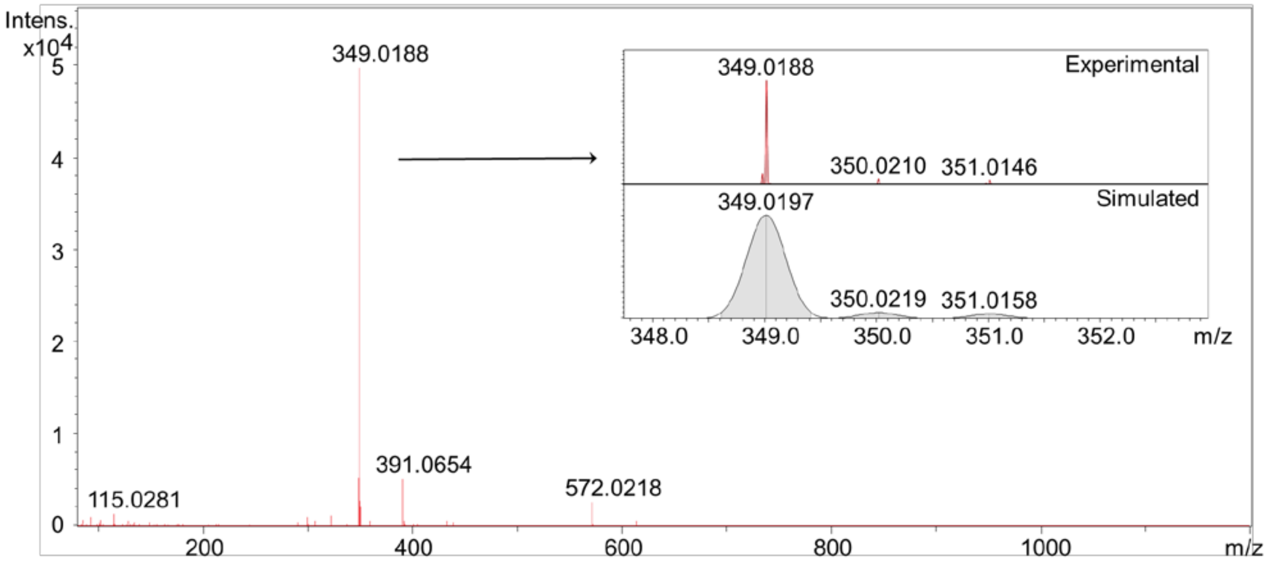

Intens.
x10^4
349.0188
391.0654
115.0281
572.0218
349.0188
Experimental
350.0210
351.0146
349.0197
Simulated
350.0219
351.0158
348.0
349.0
350.0
351.0
352.0
m/z
200
400
600
800
1000
m/z

**Au-6TG (6)**

1. 400 MHz $^{1}H$ NMR in $D_2O$

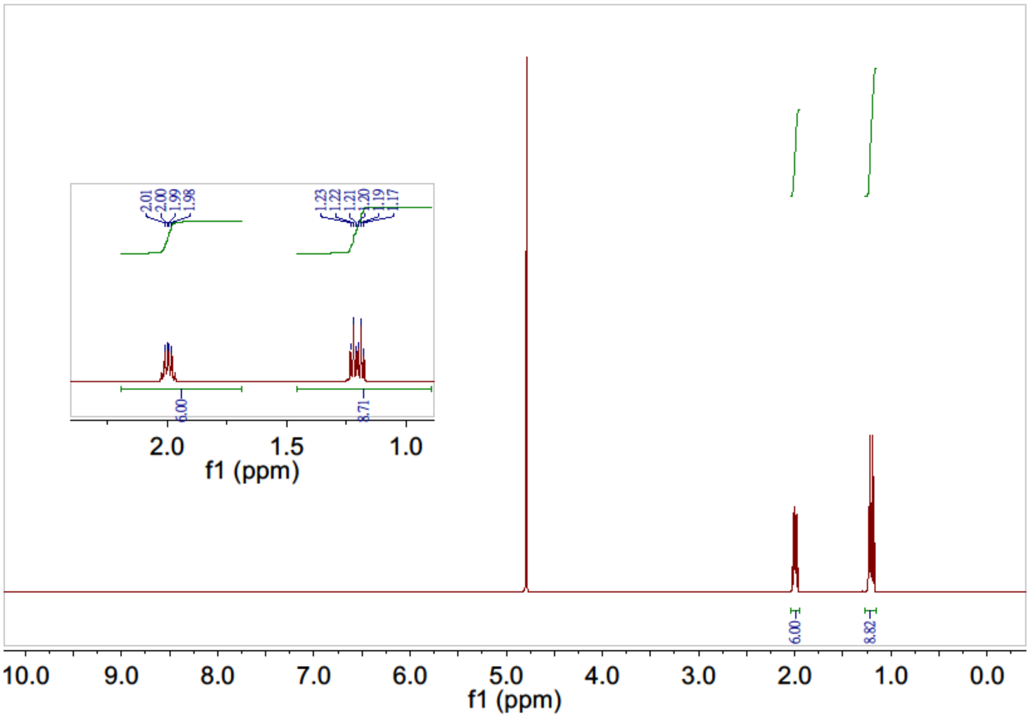


2. 400 MHz $^{13}C$ NMR in $D_2O$

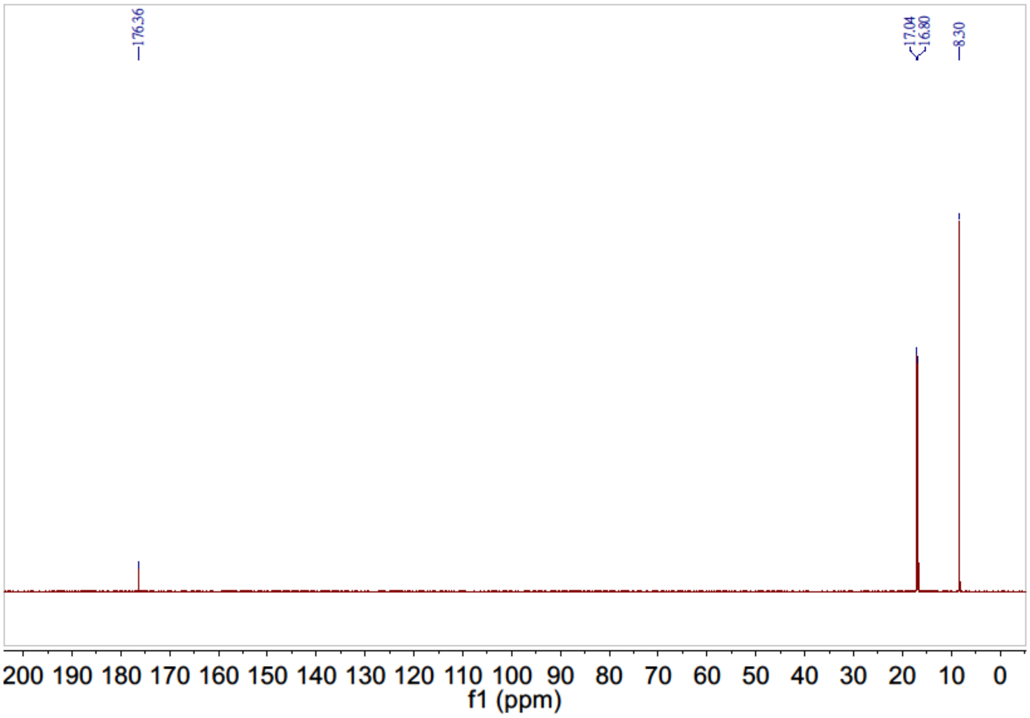

3. HR-ESI-MS

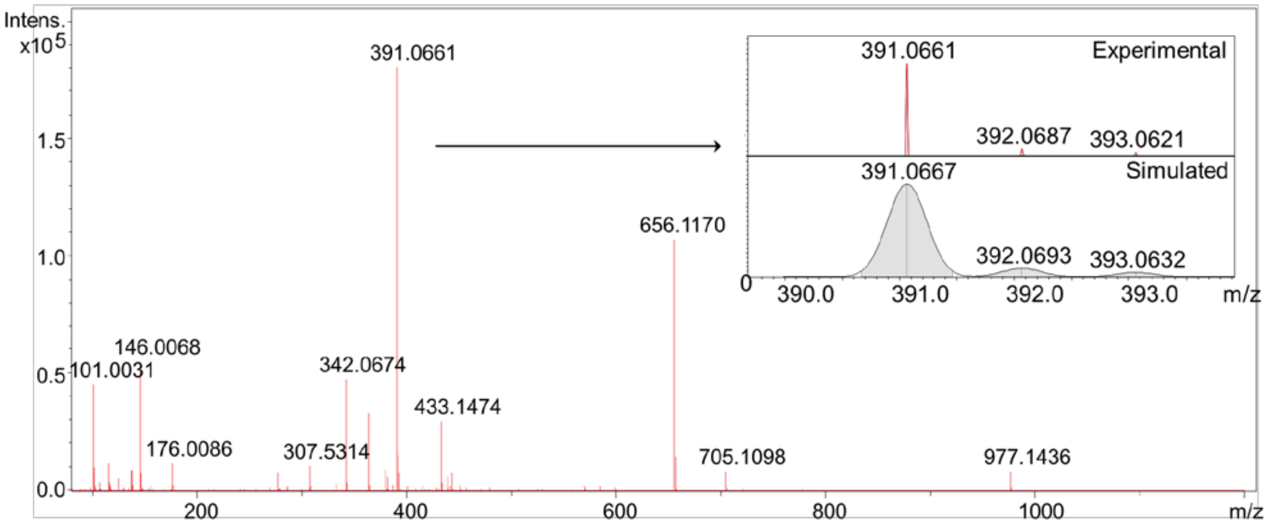
Intens.
x10^5
1.5
1.0
0.5
0.0
391.0661
656.1170
146.0068
101.0031
342.0674
433.1474
176.0086
307.5314
705.1098
977.1436
200
400
600
800
1000
m/z
391.0661
Experimental
392.0687
393.0621
391.0667
Simulated
392.0693
393.0632
0
390.0
391.0
392.0
393.0
m/z

**2-isonicotinoylhydrazine-1-carbodithioate (7)**

400 MHz $^1H$ NMR in DMSO-$d_6$

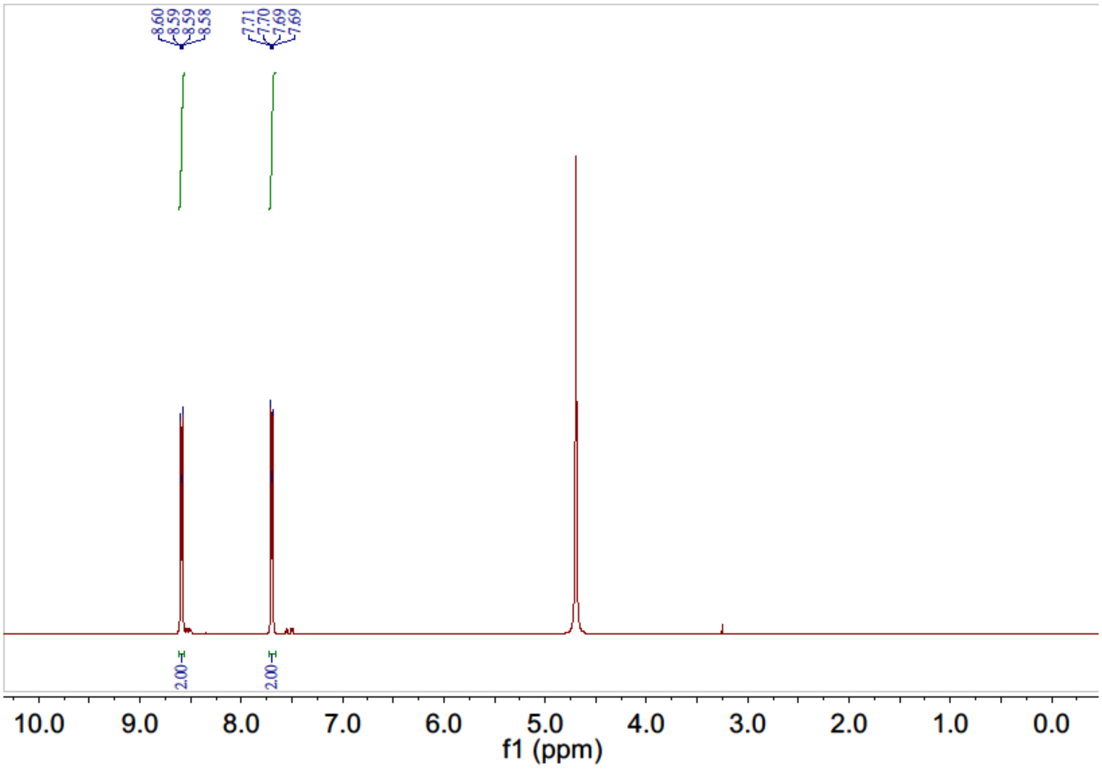

**Au-isoniazid (8)**

1. 400 MHz $^{1}$H NMR in $CDCl_3$

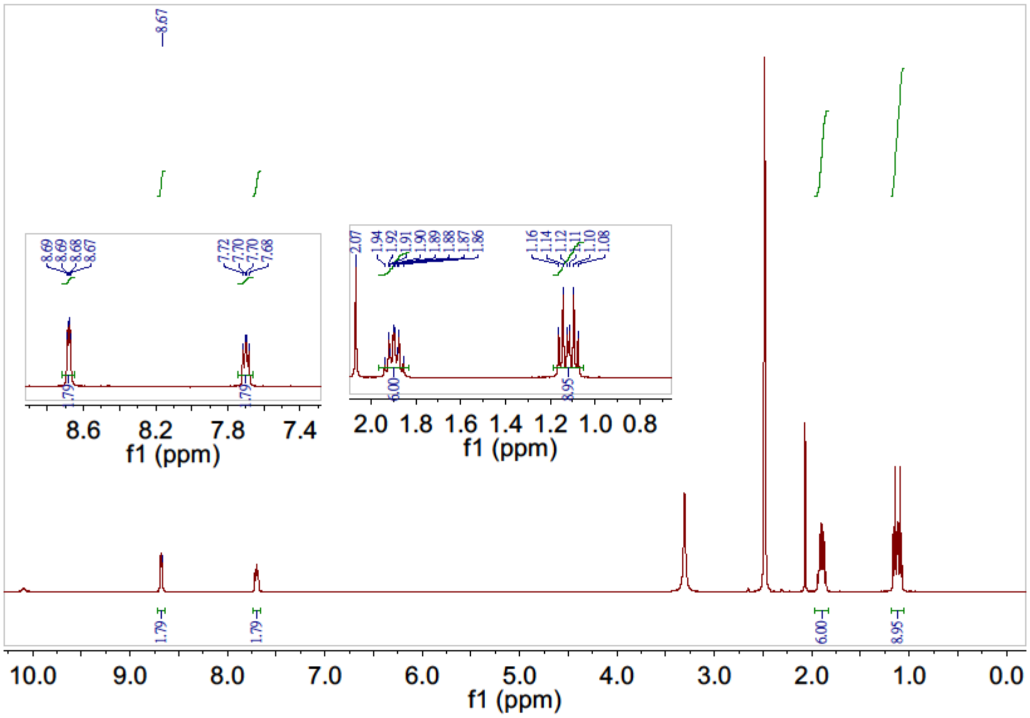


2. 400 MHz $^{13}$C NMR in $CDCl_3$

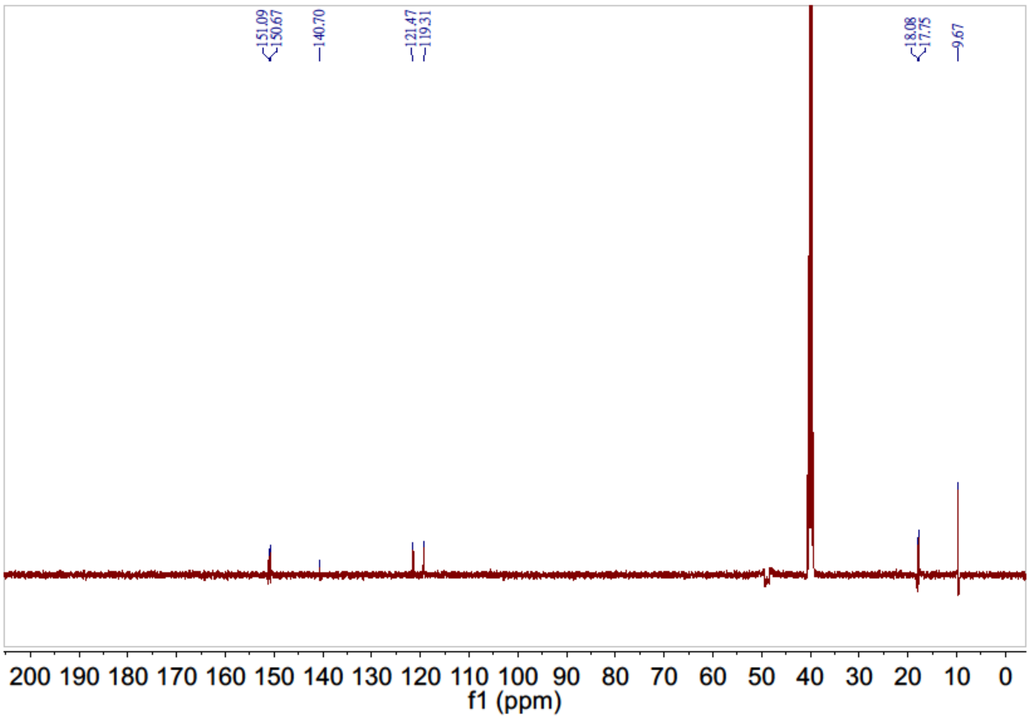

3. TGA analysis

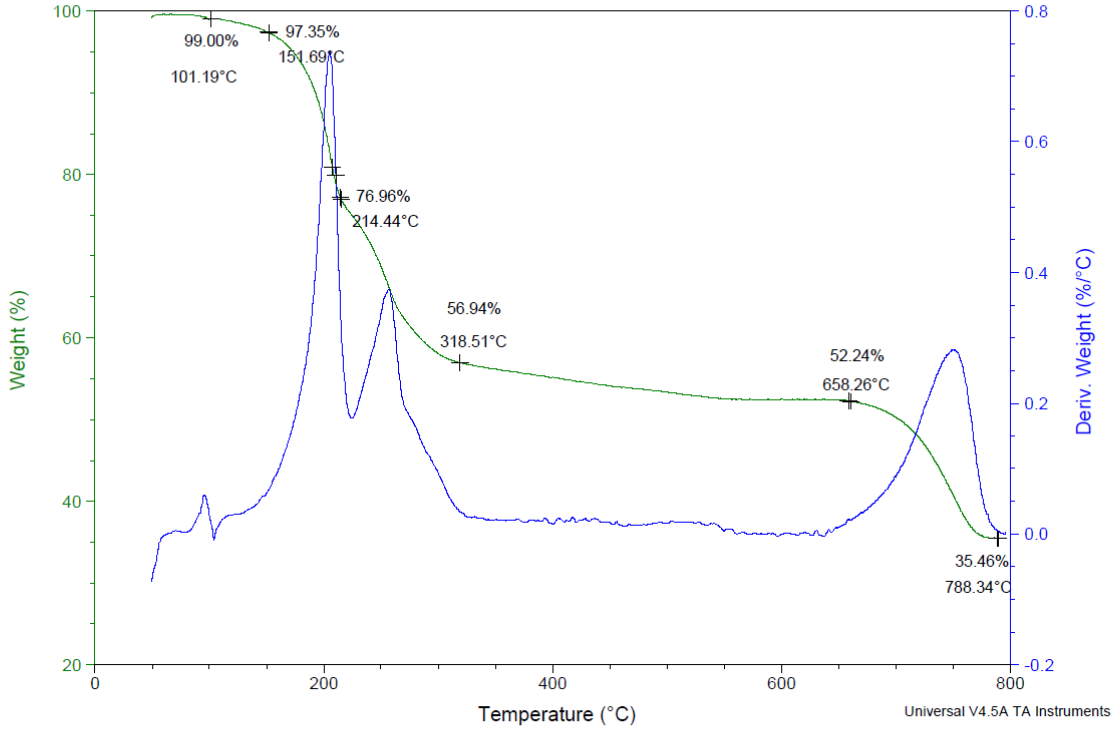


In compress air, gold(I) remains unreacted at 750°C.Proposed structure based on weight loss at 750 °C: -

[Au($PEt_3$)(isoniazid-CS2)] predicted Au content: 37.43%, founded Au content: 35.46%.

**(S)-1-((3-(3-fluoro-4-morpholinophenyl)-2-oxooxazolidin-5-yl)methyl)thiourea (9)**

400 MHz $^1$H NMR in DMSO-$d_6$

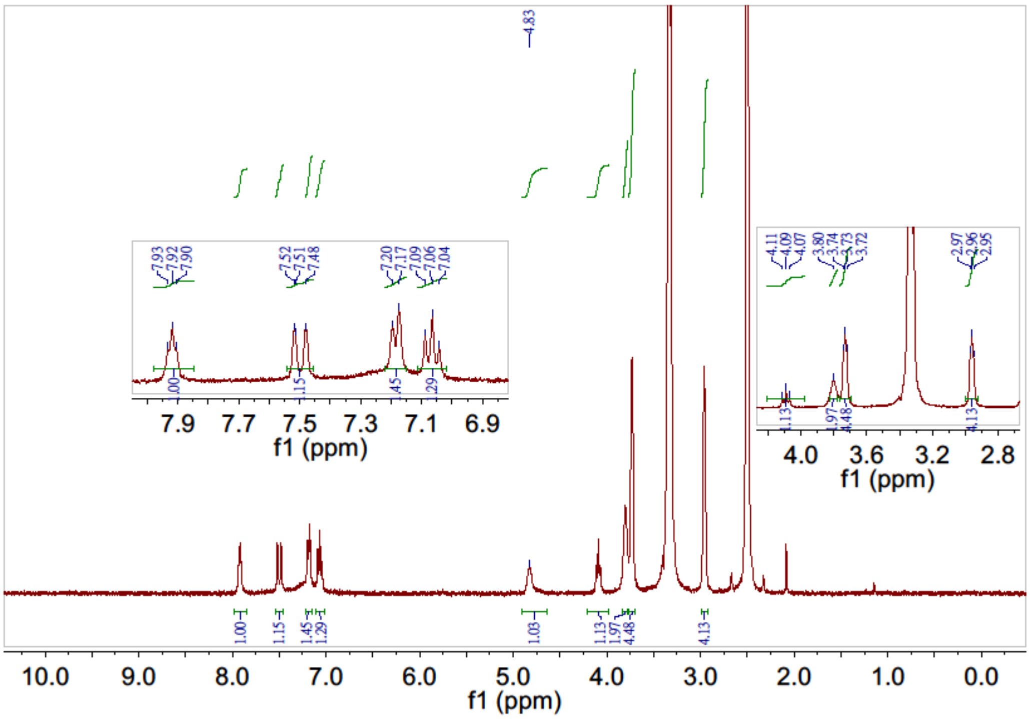

**Au-linezolid (10)**

1. 400 MHz $^{1}$H NMR in DMSO-d6

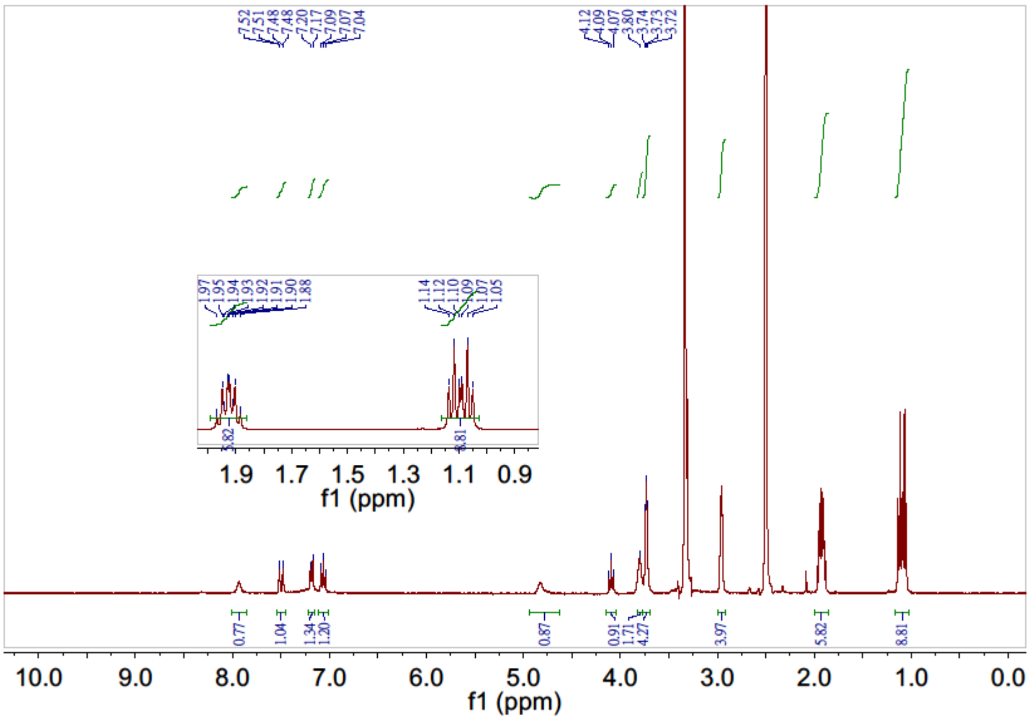

2. 400 MHz $^{13}$C NMR in DMSO-d6

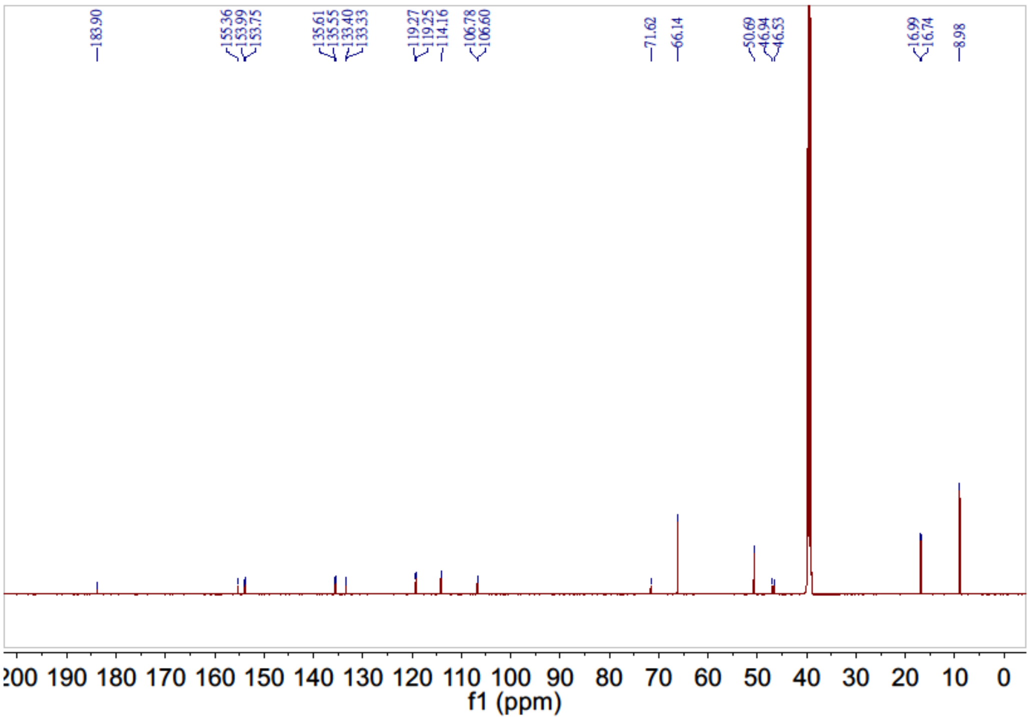

3. HR-ESI-MS

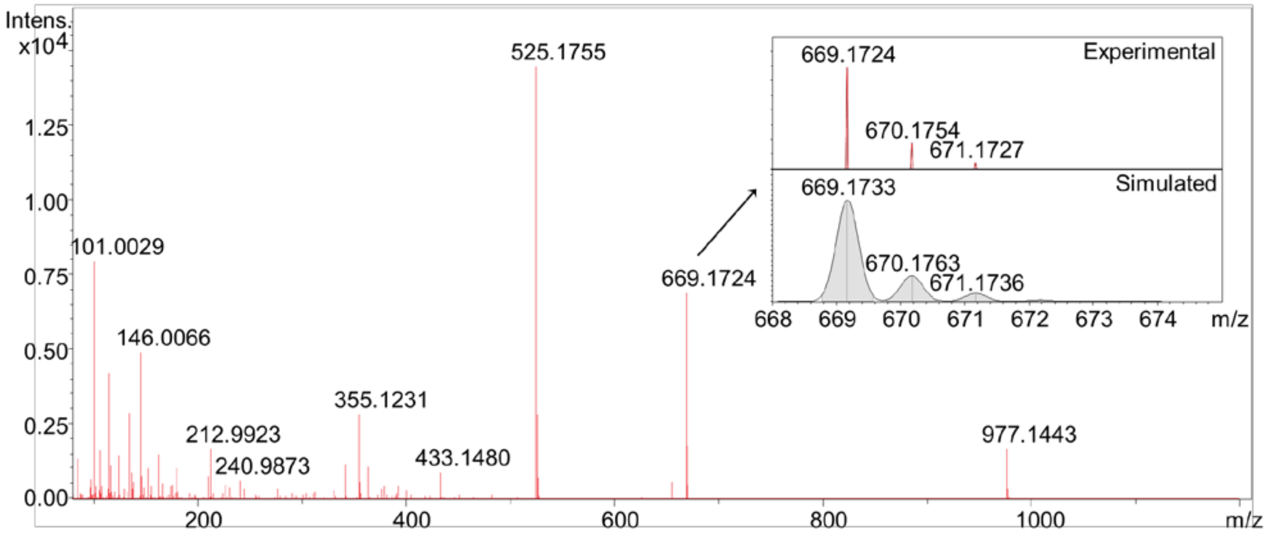
Intens.
x10^4
1.25
1.00
0.75
0.50
0.25
0.00
101.0029
146.0066
212.9923
240.9873
355.1231
433.1480
525.1755
669.1724
977.1443
200
400
600
800
1000
m/z
669.1724
Experimental
670.1754
671.1727
669.1733
Simulated
670.1763
671.1736
668
669
670
671
672
673
674
m/z

**Au-NAC (11)**

400 MHz $^1$H NMR in DMSO-d$_6$

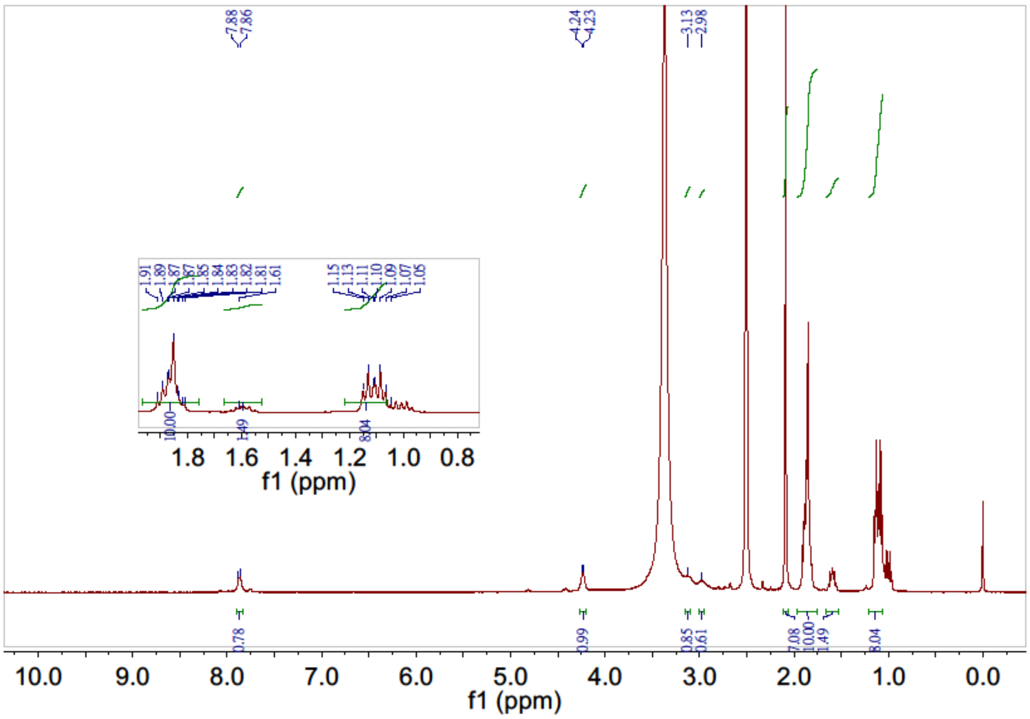

**Thiophene-Au-Cl (12)**

1. 400 MHz $^{1}H$ NMR in $CDCl_3$

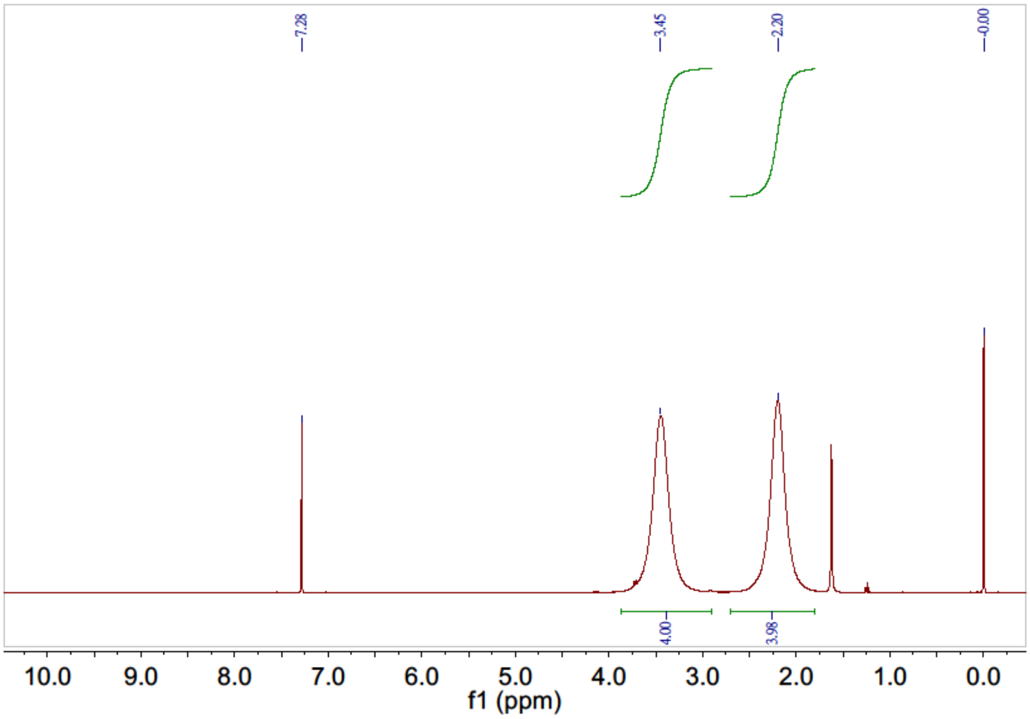


2. 200 MHz $^{13}C$ NMR in $CDCl_3$

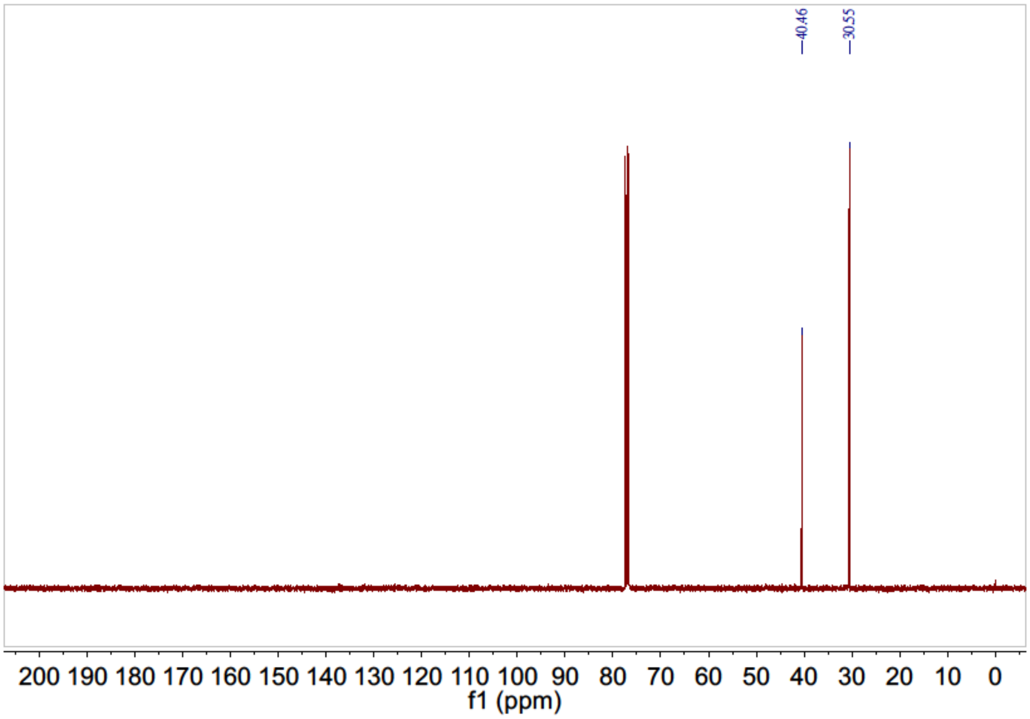

**Urotropine-Au-Cl (13)**

1. 400 MHz $^1$H NMR in DMSO-$d_6$

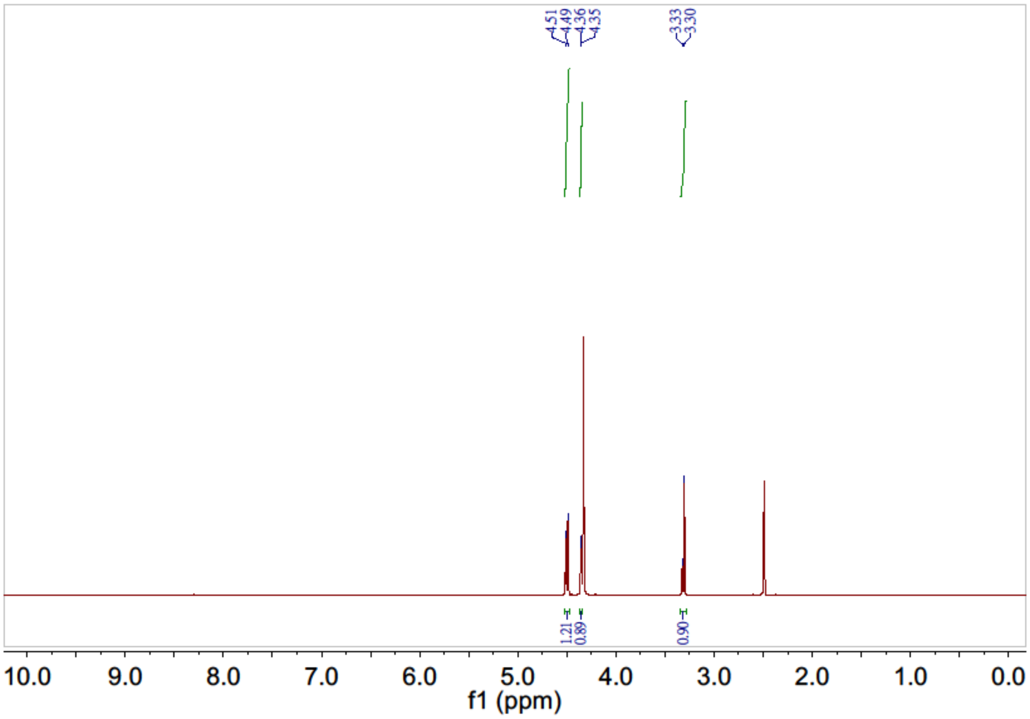


2. 400 MHz $^{13}$C NMR in DMSO-$d_6$

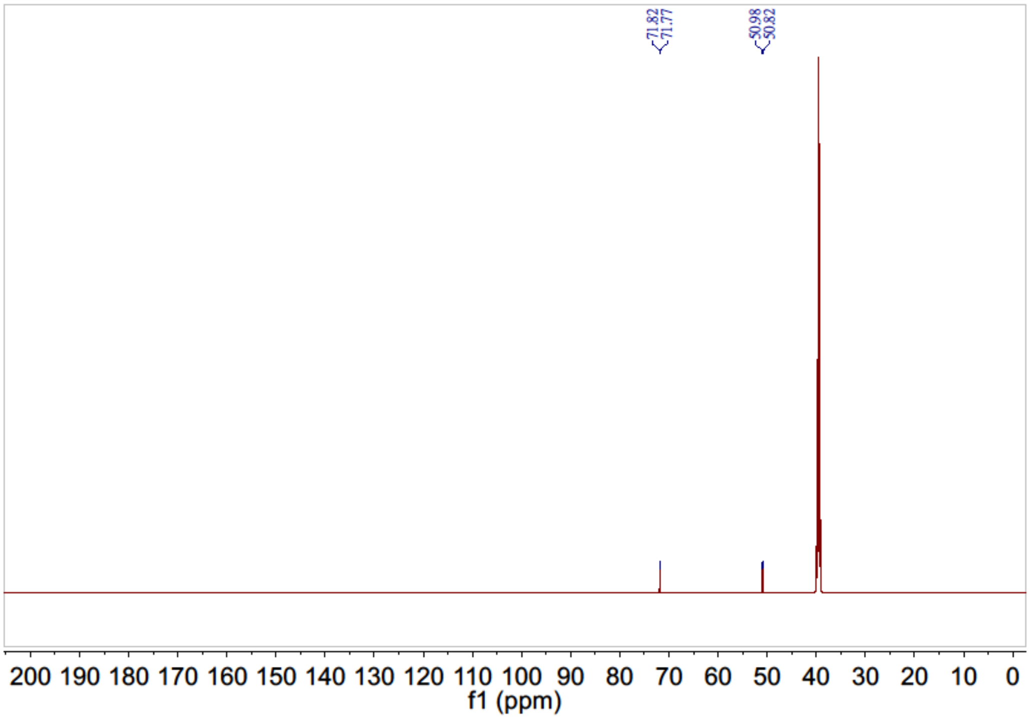

**Au-GSH (14)**

400 MHz $^{1}$H NMR in DMSO-d6

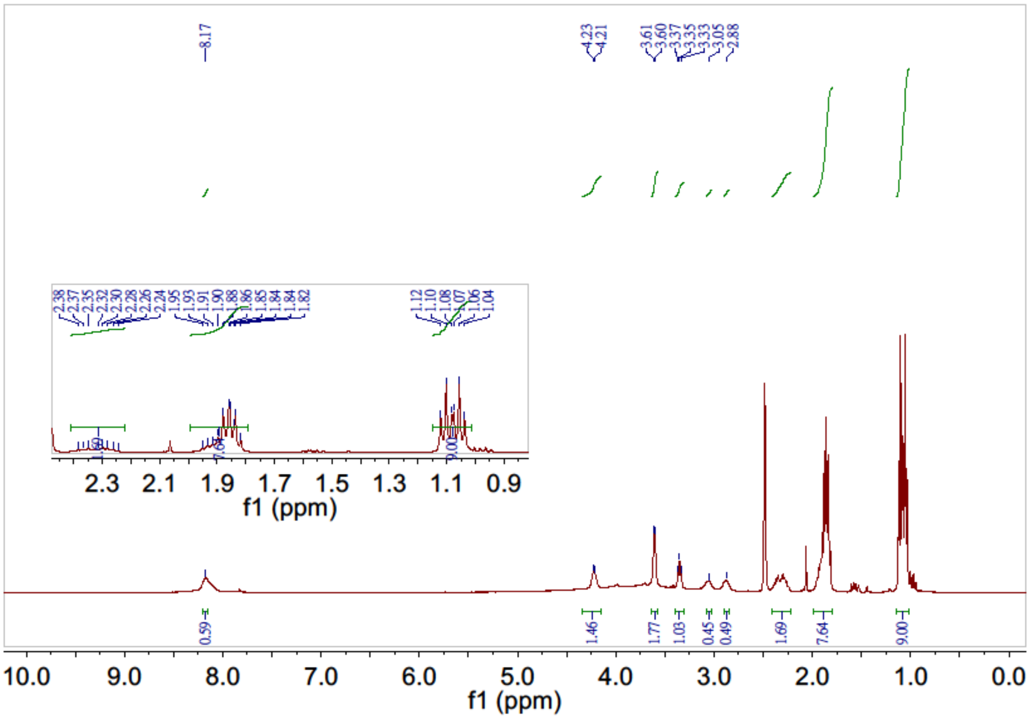

**Au-$N_2$S (15)**

1. 400 MHz $^1$H NMR in $CDCl_3$

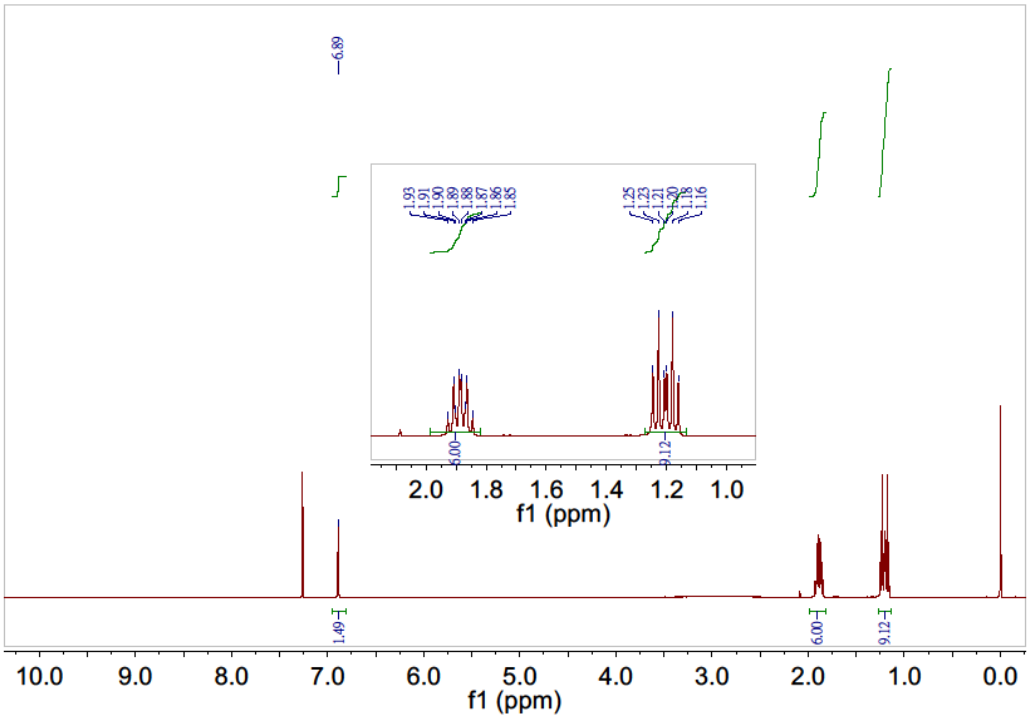


2. 400 MHz $^{13}$C NMR in $CDCl_3$

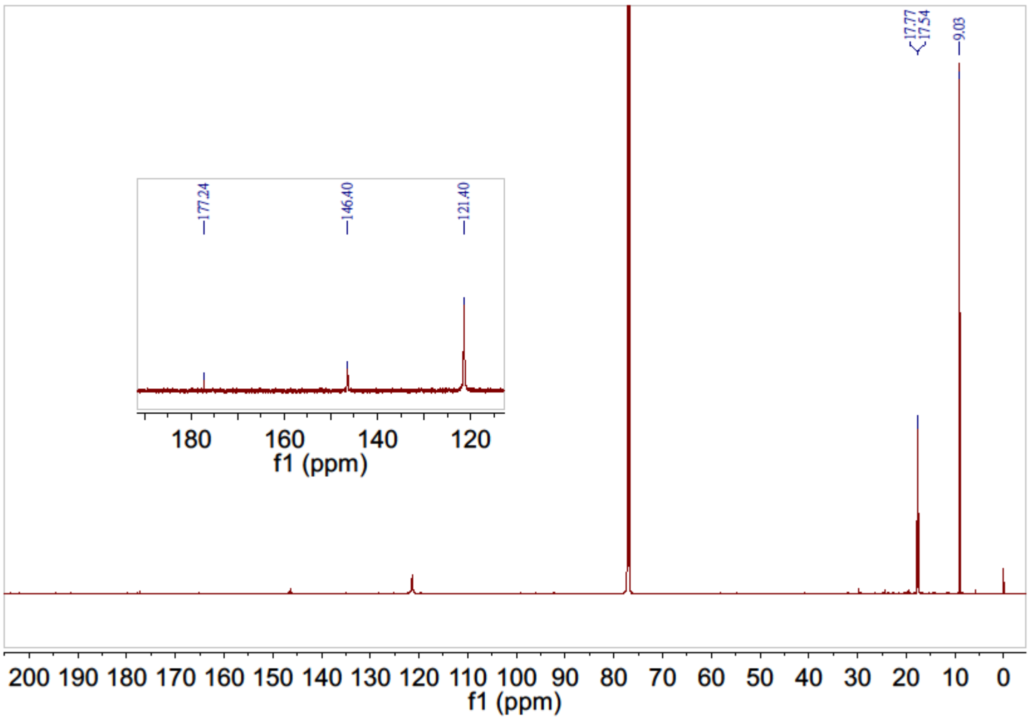

3. HR-ESI-MS

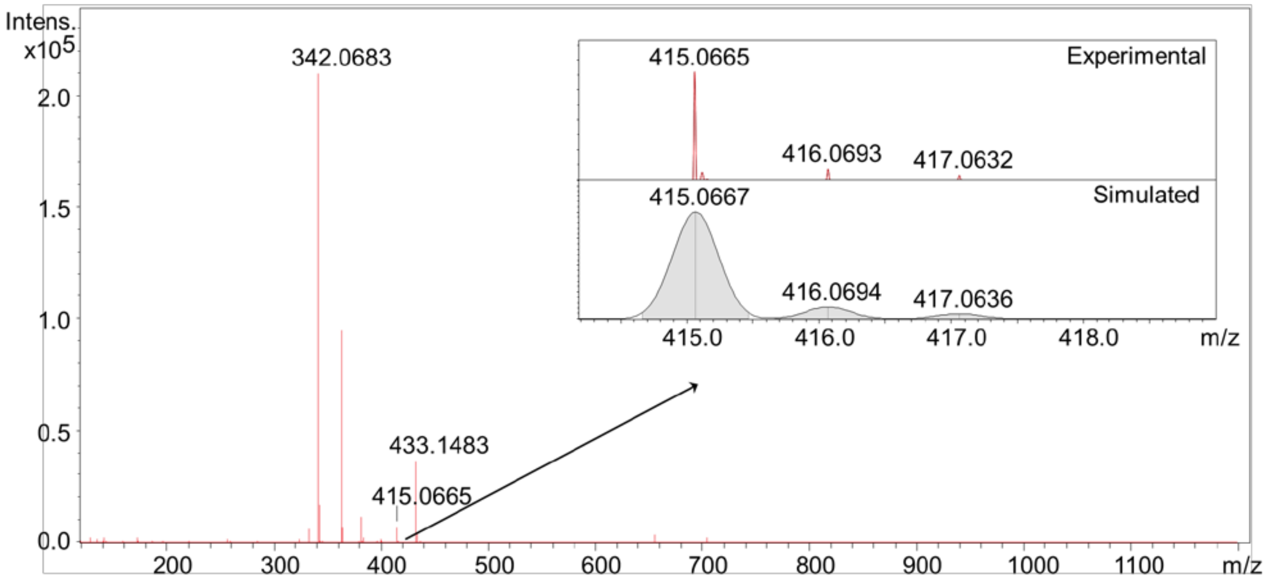

Intens.
x10^5
2.0
1.5
1.0
0.5
0.0
342.0683
433.1483
415.0665
200
300
400
500
600
700
800
900
1000
1100
m/z
415.0665
Experimental
416.0693
417.0632
415.0667
Simulated
416.0694
417.0636
415.0
416.0
417.0
418.0
m/z

**1-(9-ethyl-9H-carbazol-3-yl)thiourea (16)**

400 MHz $^1$H NMR in DMSO-d$_6$

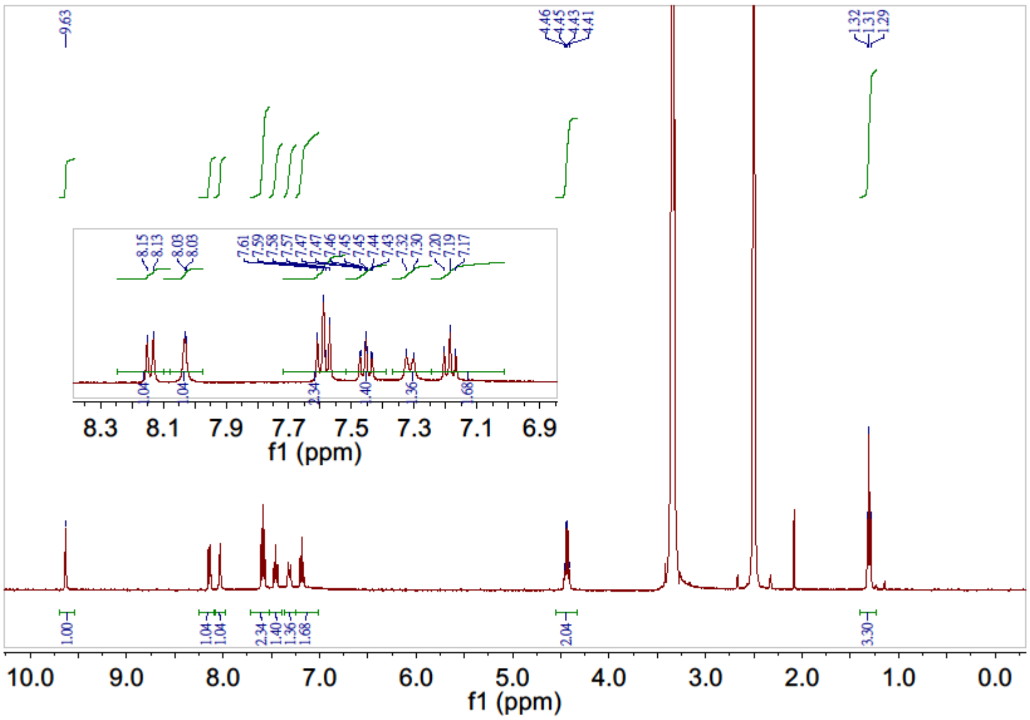

## Au-carb (17)

1. 400 MHz $^{1}H$ NMR in DMSO-$d_6$

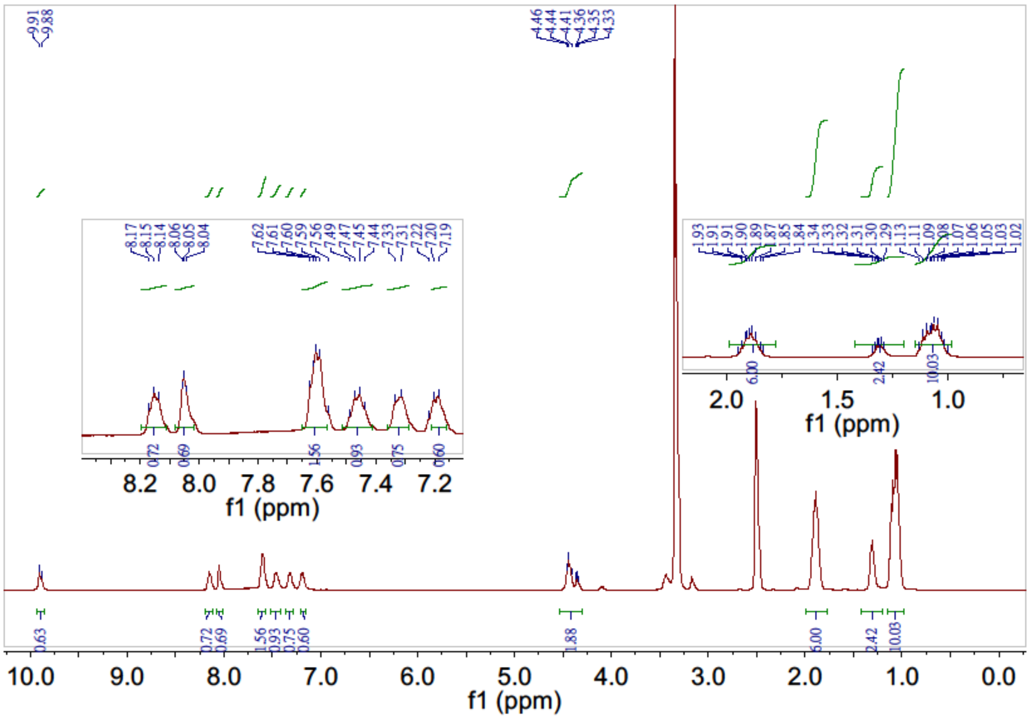


2. 400 MHz $^{13}C$ NMR in DMSO-$d_6$

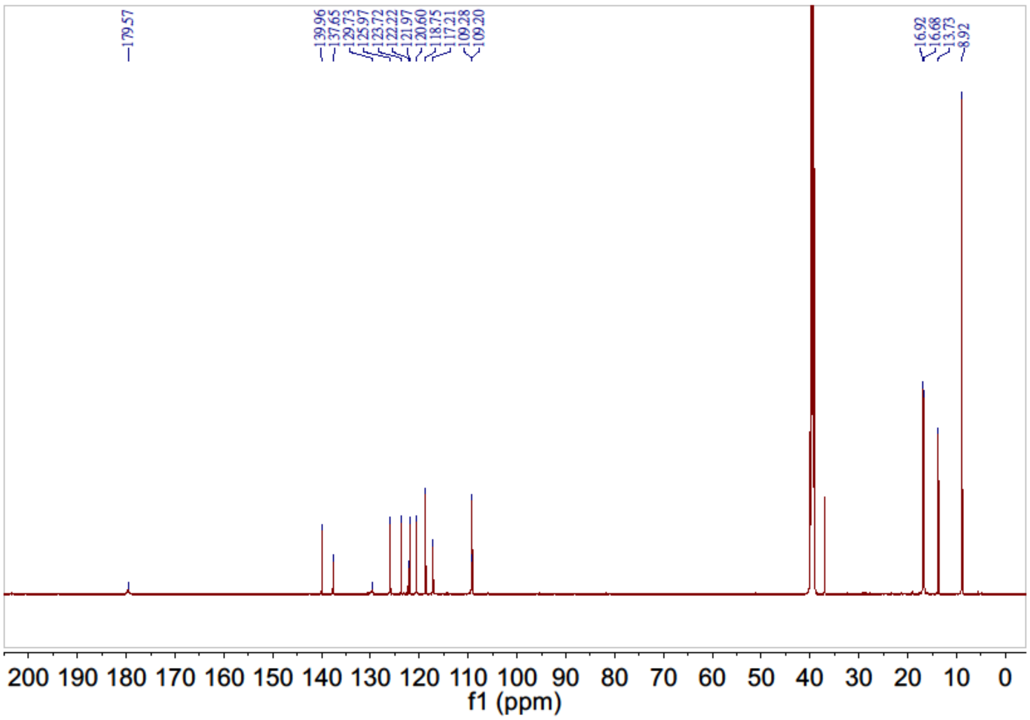

3. HR-ESI-MS

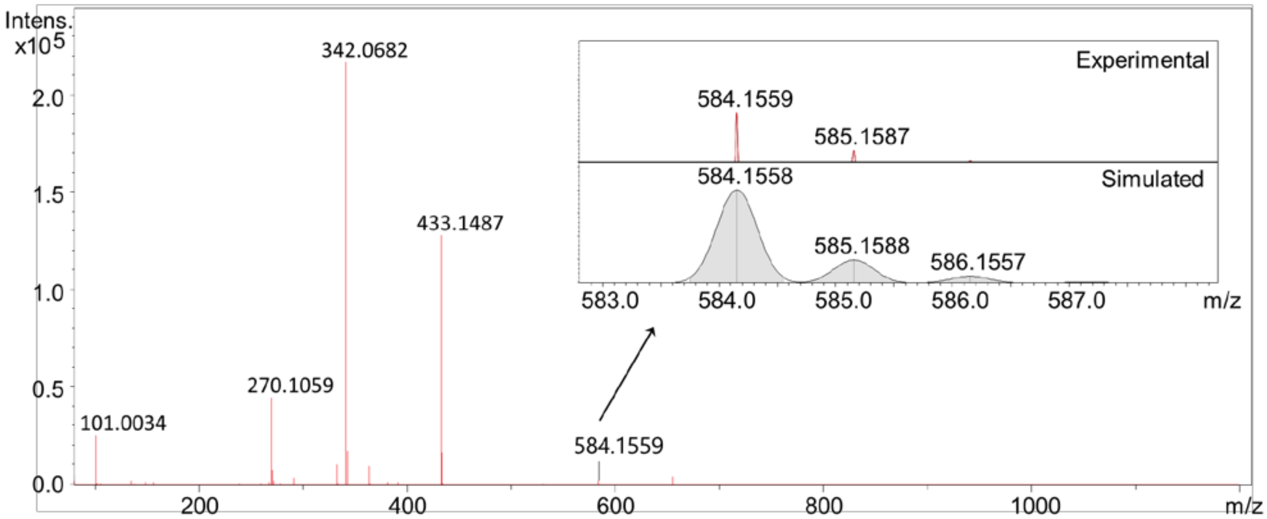
Intens.
x10^5
2.0
1.5
1.0
0.5
0.0
342.0682
433.1487
270.1059
101.0034
584.1559
200
400
600
800
1000
m/z
Experimental
584.1559
585.1587
Simulated
584.1558
585.1588
586.1557
583.0
584.0
585.0
586.0
587.0
m/z

**Au-Se (18)**

1. 400 MHz $^{1}H$ NMR in $D_2O$

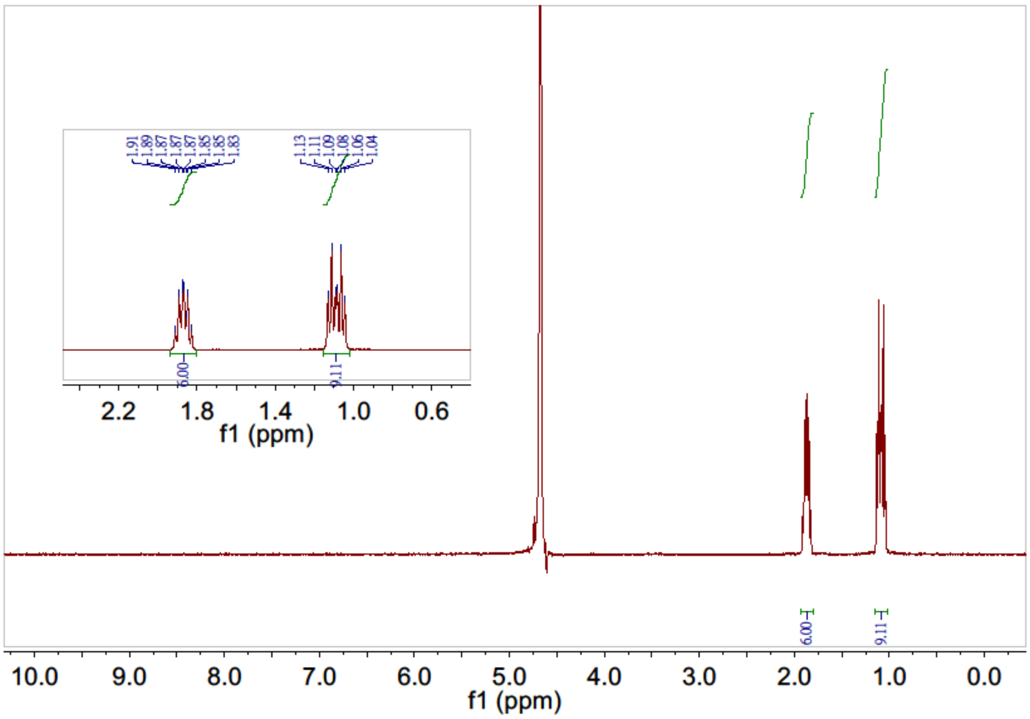


2. HR-ESI-MS

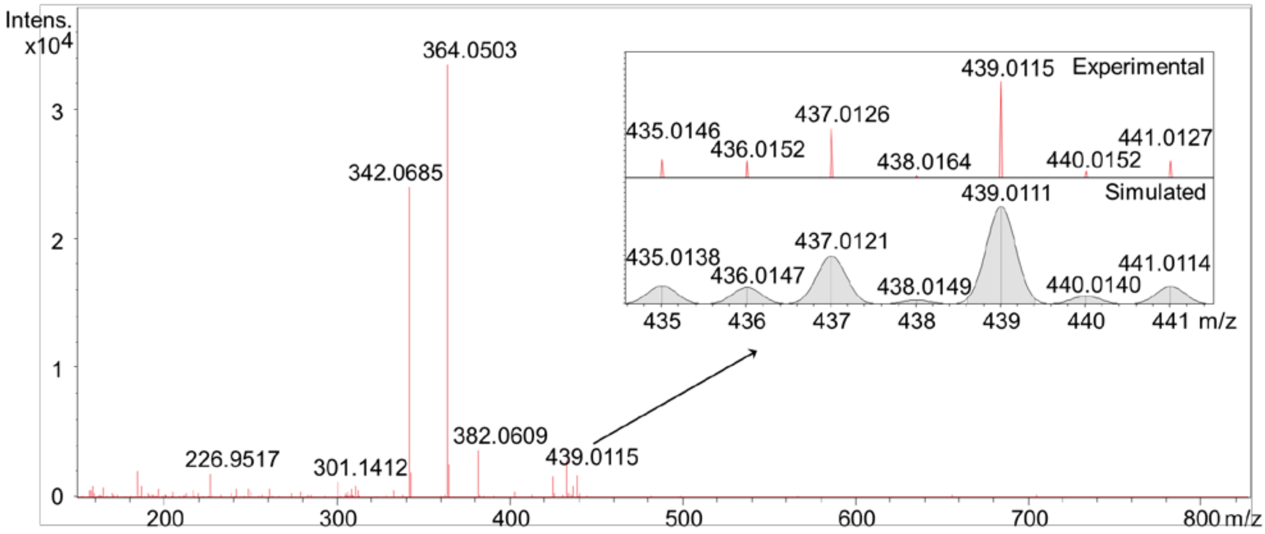

Supplement: Supplementary file 1 — Supplementary file1 (PDF 2916 KB) [file 775_2025_2118_MOESM1_ESM.pdf]
