# Supplementary figures and images for: Au(I)-based compounds inhibit nsp14/nsp10 and nsp13 (helicase) to exert anti-SARS-CoV-2 properties
Source: J Biol Inorg Chem. 2025 Jun 18;30(4-5):425–41. doi: 10.1007/s00775-025-02118-9 (PMC12316777; doi:10.1007/s00775-025-02118-9)

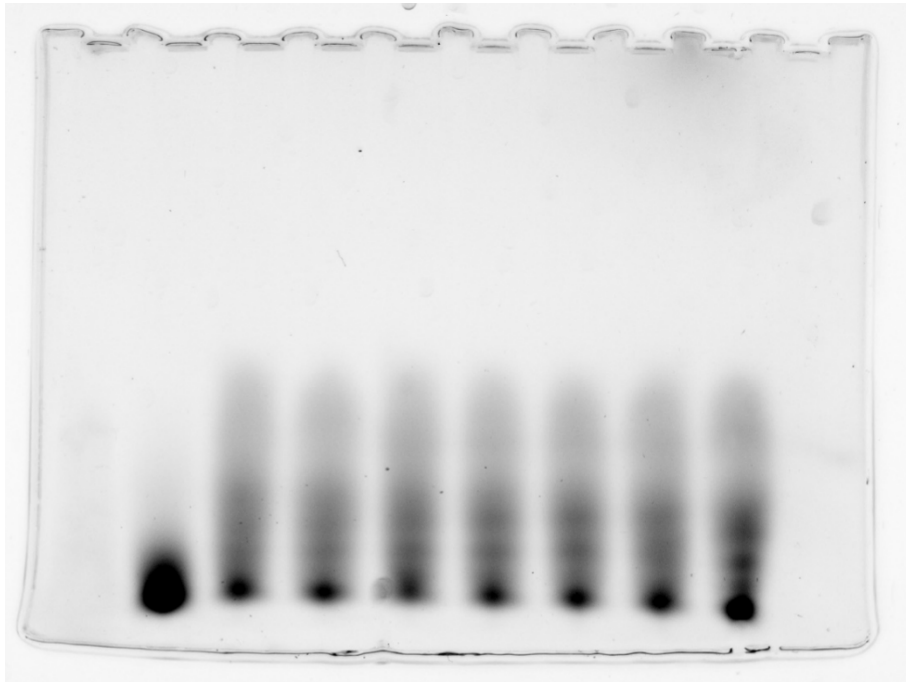

**Full uncropped gel for Fig. S2**

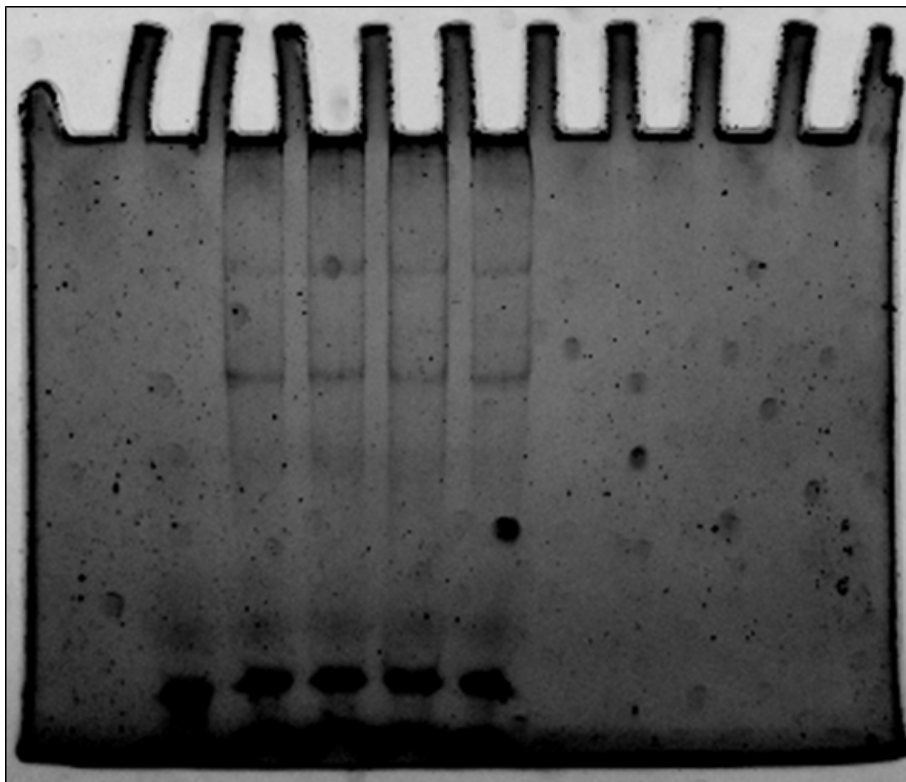

**Full uncropped gel for Fig. S3**

Supplement: Supplementary file 2 — Supplementary file2 (PDF 563 KB) [file 775_2025_2118_MOESM2_ESM.pdf]
